# Deciphering the regulation of P2X4 receptor channel gating by ivermectin using Markov models

**DOI:** 10.1371/journal.pcbi.1005643

**Published:** 2017-07-14

**Authors:** Laurent Mackay, Hana Zemkova, Stanko S. Stojilkovic, Arthur Sherman, Anmar Khadra

**Affiliations:** 1 Department of Physiology, McGill University, Montreal, Québec, Canada; 2 Department of Cellular and Molecular Neuroendocrinology, Institute of Physiology Academy of Sciences of the Czech Republic, Prague, Czech Republic; 3 Section on Cellular Signaling, *Eunice Kennedy Shiver* National Institute of Child Health and Human Development, National Institutes of Health, Bethesda, Maryland, United States of America; 4 Laboratory of Biological Modeling, National Institute of Diabetes, Digestive and Kidney Diseases, National Institutes of Health, Bethesda, Maryland, United States of America; Rutgers University, UNITED STATES

## Abstract

The P2X4 receptor (P2X4R) is a member of a family of purinergic channels activated by extracellular ATP through three orthosteric binding sites and allosterically regulated by ivermectin (IVM), a broad-spectrum antiparasitic agent. Treatment with IVM increases the efficacy of ATP to activate P2X4R, slows both receptor desensitization during sustained ATP application and receptor deactivation after ATP washout, and makes the receptor pore permeable to NMDG^+^, a large organic cation. Previously, we developed a Markov model based on the presence of one IVM binding site, which described some effects of IVM on rat P2X4R. Here we present two novel models, both with three IVM binding sites. The simpler one-layer model can reproduce many of the observed time series of evoked currents, but does not capture well the short time scales of activation, desensitization, and deactivation. A more complex two-layer model can reproduce the transient changes in desensitization observed upon IVM application, the significant increase in ATP-induced current amplitudes at low IVM concentrations, and the modest increase in the unitary conductance. In addition, the two-layer model suggests that this receptor can exist in a deeply inactivated state, not responsive to ATP, and that its desensitization rate can be altered by each of the three IVM binding sites. In summary, this study provides a detailed analysis of P2X4R kinetics and elucidates the orthosteric and allosteric mechanisms regulating its channel gating.

## Author summary

Ligand-gated ion channels play a crucial role in controlling many physiological and pathophysiological processes. Deciphering the gating kinetics of these channels is thus fundamental to understanding how these processes work. ATP-gated purinergic P2X receptors (P2XRs) are prototypic examples of such channels. They are ubiquitously expressed and play roles in numerous cellular processes, including neurotransmission, inflammation, and chronic pain. Seven P2X subunits, named P2X1 through P2X7, and several splice forms of these subunits have been identified in mammal. The receptors are organized as homo- or heterotrimers, each possessing three ATP-binding sites that, when occupied, lead to receptor activation and channel opening. The P2XRs are non-selective cation channels and the gating properties differ between the various receptors. Previously, we have used biophysical and mathematical modeling approaches to decipher the kinetics of homomeric P2X2aR, P2X2bR, P2X4R, and P2X7R. Here we extended our work on P2X4R gating. We developed two mathematical models that could capture the various patterns of ionic currents recorded experimentally and explain the particularly complex kinetics of the receptor during orthosteric activation and allosteric modulation. This was achieved by designing a computationally efficient, inference-based fitting algorithm that allowed for parameter optimization and model comparisons.

## Introduction

Purinergic P2X receptors (P2XRs) are a family of ligand-gated non-selective cation channels that are activated by extracellular adenosine 5'-triphosphate (ATP). In mammals, there are seven distinct subunits of this family of proteins, labeled P2X1-7. Each subunit contains intracellular N- and C-termini connected to the first and second transmembrane (TM) domains, respectively, followed by a large extracellular loop commonly referred to as the ectodomain. It is also well established that P2X subunits aggregate to form functional trimers [1–5]; receptors may be composed of either one type of subunit (homotrimer) or a mixture of more than one type of subunit (heterotrimer) [6]. When coordinated in a trimer, the interfaces between adjacent ectodomains form three binding pockets for ATP [7]. These ectodomains also form fenestrations which are lined by negatively charged amino acids that attract cations, and the cation selectivity of these channels is determined by the selectivity filter localized in the TM2 domain [8].

The binding of two or three ATP molecules to the extracellular binding sites induces conformational changes in the ectodomain and subsequently the TM domains, causing the channel opening. The gating of P2XR by ATP and other orthosteric agonists can be broken down into three distinguishable phases, activation, desensitization, and deactivation, defined by their ionic current kinetics in whole-cell recordings [9, 10]. Activation is a rapid phase of channel opening that corresponds to increasing inward current subsequent to agonist application. This is usually followed by the desensitization phase, a decay of current amplitude in the maintained presence of an agonist, with an onset that is slower than that of activation. After agonist removal from the medium, a relatively rapid decrease in current amplitude, referred to as the deactivation phase, is observed. Receptors differ in both their sensitivity to agonists and the kinetics of the phases (with desensitization being transient and controversial in P2X7Rs) [9, 11].

It has also been suggested that P2X2R and P2X7R are capable of exhibiting another phase in their gating, termed dilation, when the receptor pore was thought to progressively enlarge during sustained ATP application. Two observations were used as evidence for pore dilation: the ability of these receptors to become permeable to N-methyl-D-glucamine (NMDG^+^), a large organic cation (~7.3 Å in mean diameter), and the change in reversal potential (E_rev_) during a ramp protocol, when cells were bathed in a medium containing only NMDG^+^ with pipette containing only NaCl [12]. Recent investigations, however, have shown that changes in E_rev_ during prolonged channel activation of P2X2R do not reflect pore dilation, but rather time-dependent alterations in the concentration of intracellular ions, specifically washout of intracellular Na^+^ and gain of NMDG^+^ through the initially opened large pore of P2XR [13, 14] and that permeation of NMDG^+^ can also occur without pore dilation [15].

In addition to orthosteric regulation, P2XRs also exhibit allosteric regulation [9]), as is evident from the action of ivermectin (IVM) on P2X4R channels. Extracellularly applied IVM increases current amplitude at low concentrations, and increases the sensitivity of receptors to ATP and partial agonists at higher concentrations. IVM also decreases the extent of desensitization in the continuous presence of agonist and prolongs deactivation of the receptor after the removal of agonists [16–18]. Furthermore, P2X4R is not substantially permeable to NMDG^+^ natively [16, 19], but displays a shift in E_rev_ in the presence of IVM, suggesting that the channel pore is permeable to NMDG^+^ in IVM-treated cells [20].

The action of IVM on P2X4R gating is also time-dependent; i.e. the cells must be exposed to IVM for at least 30 s, in the absence of ATP, to alter the P2X4R gating (compared to ms for orthosteric activation) [20]. The onset of IVM’s potentiating effect on P2X4R current amplitude is faster than the effects of IVM on deactivation kinetics [17, 21]. Consequently it has been postulated that the two distinct effects of IVM are due to binding at two distinct sites [17]. Experiments with chimeric receptors containing domains from IVM-sensitive P2X4R and the IVM-insensitive P2X2R have provided evidence that TM domains play a critical role in this allosteric modulation by IVM [18]. The location of the IVM binding site has not yet been addressed in the context of the recent crystal structures of a zfP2X4R [2, 3]. However, IVM apparently inserts between pairs of neighboring subunits of the P2X4R channel in the membrane and interferes with the molecular rearrangement in the TM domains involved in channel gating, similarly to glutamate-gated chloride channels crystalized with IVMs [22]. Accordingly, there should be three potential binding sites for IVM in the P2X4R, as there are three clefts between subunits. Such a topography of IVM binding sites provides rationale as to why receptors (not previously stimulated orthosterically) must be exposed to IVM for a prolonged period for it to be effective. Subsequently, we have used the term priming to describe the time and concentration dependence of IVM to occupy its binding sites and the resulting development of its varied allosteric effects.

In recent years, mathematical modeling has begun to shed light on many aspects of P2XRs and to guide experimental designs to arrive at a more complete understanding of channel gating [19, 20, 23–25]. Biophysically detailed Markov models that describe individual orthosteric binding sites and their allosteric modulation, have been very successful in deciphering the kinetics of P2X homotrimers and succinctly explaining many phenomena [19, 20, 24–26]. They consider important biophysical details such as the conformational states of individual binding sites and other structural components of the receptor. One of these models for P2X4R is a simple Markov model that takes into account the sequential binding of ATP to its three subunits and assumes that IVM causes receptor sensitization upon the binding of three ATP molecules, that all ATP unbinding rates are decreasing functions of IVM concentration, and that IVM induces a change in ion selectivity caused by the assumed pore dilation [20]. In this model, the three allosteric effects of IVM on P2X4Rs are induced by a single IVM-dependent transition that allowed for generating the shift in E_rev_ during the ramp protocol. However, the published model is unable to account for effects of pre-treatment with IVM before ATP application. The model also predicts a large (> 150%) increase in the unitary (single-channel) conductance of the receptor, in contrast to experimental evidence [17] indicating that there is at most 20% increase in unitary conductance.

To satisfy these constraints, we developed two substantially larger models that not only fit the data more closely in more experimental circumstances but offer better insights into how the kinetics of ATP and IVM sequential binding to P2X4R affect P2X4R activation, desensitization, and deactivation. They also illustrate how changes in ion selectivity of these receptors are manifested, as well as predict the previously unappreciated existence of receptor states (including the deeply inactivated and primed states) that are not directly observable in the experimental current recordings.

## Results

### IVM reduces P2X4R pore selectivity

When HEK293 cells expressing rat P2X4R were bathed in Ca^2+^-containing medium, where Na^+^ was substituted by NMDG^+^ and a voltage ramp from −80 mV to +80 mV was applied to the cell, E_rev_ was not found to change during sustained applications of 100 μM concentrations of ATP (S1 Fig, left). However, pretreatment with 3 μM IVM for 60 s caused a positive shift in E_rev_ during sustained applications of 100 μM ATP (S1 Fig, right). This is consistent with our earlier work [20] and the finding of others that IVM potentiates ATP-induced responses and increases permeability for NMDG^+^, but cannot activate P2X4R channels on its own [16]. Because strategies that rely on changes in E_rev_ to provide evidence for large pore formation during sustained stimulation with agonist were questioned [13, 15], we examined currents induced by ATP in Ca^2+^-free/NMDG^+^-containing medium. S2 Fig shows that in the absence of Ca^2+^, a 40-s application of ATP (100 μM) at −60 mV in bi-ionic NMDG^+^ out/Na^+^ in solution (where the reversal potential of ATP-induced current is about -70 mV [27]) evoked only outward Na^+^ current, whereas in the presence of IVM, outward Na^+^ current was followed by inward NMDG^+^ current. These experiments do not argue against findings with P2X2R presented recently [13] but provide evidence for the existence of two conductive pore states of P2X4R. These pore states, termed open_1_ and open_2_, differ in their selectivity for organic cations (a consequence of altered relative permeability *P*_*NMDG*_/*P*_*Na*_), and the priming of receptors by IVM is needed to switch from one conductive state to another.

We will demonstrate later that the shift in E_rev_ does not require an increase in unitary conductance associated with the open_2_ state(s), but rather depends on the selectivity associated with Na^+^ and NMDG^+^, as suggested by the Goldman-Hodgkin-Katz equation,

$${\text{V}}_{\text{rev}}=\frac{RT}{F}\text{ln}\frac{P_{\text{Na}}{\left[{\text{Na}}^{+}\right]}_{\text{out}}\;+\;P_{\text{NMDG}}{{\text{[NMDG}}^{\text{+}}\text{]}}_{\text{out}}}{P_{\text{Na}}{\left[{\text{Na}}^{+}\right]}_{\text{in}}\;+\;P_{\text{NMDG}}{{\text{[NMDG}}^{\text{+}}\text{]}}_{\text{in}}},$$

where *R* is the gas constant, *T* is the absolute temperature, *F* is Faraday’s constant, *P*_Na_(*P*_NMDG_) is Na^+^ (NMDG^+^) permeability, and [Na^+^]_out_ ([Na^+^]_in_) is Na^+^ concentration outside (inside) the cell, whereas [NMDG^+^]_out_ ([NMDG^+^]_in_) is NMDG^+^ concentration outside (inside) the cell. Because the experimentally observed E_rev_ shift is independent of the increase in unitary conductance, the term open_2_ state will be used to refer to both the (small) increase in unitary conductance and the (large) change in ion selectivity of the P2X4R pore.

### Desensitization masks the increase in unitary conductance in P2X4R

The previous paragraph proposed that P2X4R opens with the open_2_ pore state(s) in the presence of IVM, which may have an increase in unitary conductance of as much as 20% [17]. At the same time, the ramp protocol shows a decrease in the slopes of the I-V curves (S1 Fig). To enforce such an outcome in any potential model of P2X4R with IVM-dependent allosteric transitions between open states, we require the rate of increase in the probability of open states due to allostery (open_1_ → open_2_) to be slower than the rate of decrease in the probability of open states (open_1_ → desensitized). We propose that the increased conductance of the open_2_ state(s) of the receptor pores is masked by desensitization in a time-dependent manner, similar to our previous finding with P2X2R [19]. We are thus led to assume that the probability of finding open receptors on the cell membrane, *P*(open_1_), is a strictly decreasing function of time $\left(\dot{P}({\text{open}}_{1})\;<\;0\right)$. On the other hand, we expect that the probability of finding a receptor whose pore is in the open_2_ state(s), *P*(open_2_) to be an increasing function of time $\left(\dot{P}({\text{open}}_{2})\;>\;0\right)$.

Without specifying a Markov model to describe P2X4R kinetics, we may consider a generic equation for current production in these receptors, capable of distinguishing open_1_ and open_2_ states based on their conductances and reversal potentials established after washout of intracellular Na^+^ and gain of NMDG^+^. According to the description above, we can write the equation for current as

$$I=g_{1}P({\text{open}}_{1})(V-E_{1})+g_{2}P({\text{open}}_{2})(V-E_{2})$$ (1)

where *g*_1_ is the maximum conductance of the open_1_ state(s), *g*_2_ (> *g*_1_) is the maximum conductance of open_2_ state(s), and *E*_1_ and *E*_2_ are the reversal potentials associated with the open_1_ and open_2_ states, respectively. The current equation can be rewritten in a standard form to isolate the total conductance and reversal potential of the cell, as follows

$$I=g_{tot}(V-E_{tot}),$$ (2)

where *g*_*tot*_ and *E*_*tot*_ are the total conductance and reversal potential of the cell, respectively. By equating Eqs (1) and (2), we obtain

$$g_{tot}=g_{1}P({\text{open}}_{1})+g_{2}P({\text{open}}_{2}).$$

The requirement for the slope of the I-V curves to decrease during the ramp protocol can be met if the total conductance of the receptor population decreases over time, i.e., ${\dot{g}}_{tot}<0$. Taking the time derivative of *g*_*tot*_ and rearranging the terms, we obtain

$${\dot{g}}_{tot}=g_{1}\left(\dot{P}({\text{open}}_{1})+\frac{g_{2}}{g_{1}}\dot{P}({\text{open}}_{2})\right),$$

which is strictly negative if we impose the condition

$$-\dot{P}({\text{open}}_{1})>\frac{g_{2}}{g_{1}}\dot{P}({\text{open}}_{2}).$$ (3)

It follows that

$$\left|\dot{P}({\text{open}}_{1})\right|>\frac{g_{2}}{g_{1}}\dot{P}({\text{open}}_{2}).$$

This result implies that the total conductance of the cell will decrease if the fraction of open receptors decreases more rapidly than the ratio of the open_1_-to-open_2_ maximum conductances times the rate of increase of the open_2_ state(s). Thus, in order to capture the decrease in the slopes of the I-V curves in any model development, we have to increase the rate of desensitization of the open states, reduce the rate of increase of open_2_ state(s) or decrease the ratio between the open_2_ and open_1_ conductances.

As a first approximation, we can attribute the decrease in the fraction of open states to two processes, desensitization and priming of receptors, related by the equation

$$-\dot{P}({\text{open}}_{1})=\dot{P}({\text{open}}_{2})+\delta$$ (4)

where ***δ*** is the rate of change of open receptors due to desensitization. Furthermore, letting *g*_2_ satisfy

$$g_{2}=g_{1}\left(1+f\right)$$

where *f* is the fractional increase in unitary conductance, we can substitute this expression into Eq (3) to obtain

$$\delta >f\dot{P}({\text{open}}_{2}).$$ (5)

Inequality Eq (5) represents a new condition that can be used to produce the decrease in total conductance seen in the ramp protocol. For example, if we consider the experimentally observed value of ~0.2 for *f* in human P2X4R, then the rate of desensitization only needs to be one fifth the rate of the IVM-induced unitary conductance increase in order to mask its effect on the slopes of the I-V curves.

The desensitization rate of naïve receptors is well characterized by the current recordings produced during prolonged application of ATP, which can be used to constrain ***δ*** as a fixed parameter. The IVM-induced increase in unitary conductance has not been determined for rat P2X4R, nor its time-course. We determine these in the Markov model (discussed below) by fitting the total current, imposing Inequality Eq (5) to ensure that the total conductance of the cell decreases during the ramp protocol (due to desensitization).

### IVM affects receptor desensitization and deactivation kinetics

**Fig 1:**
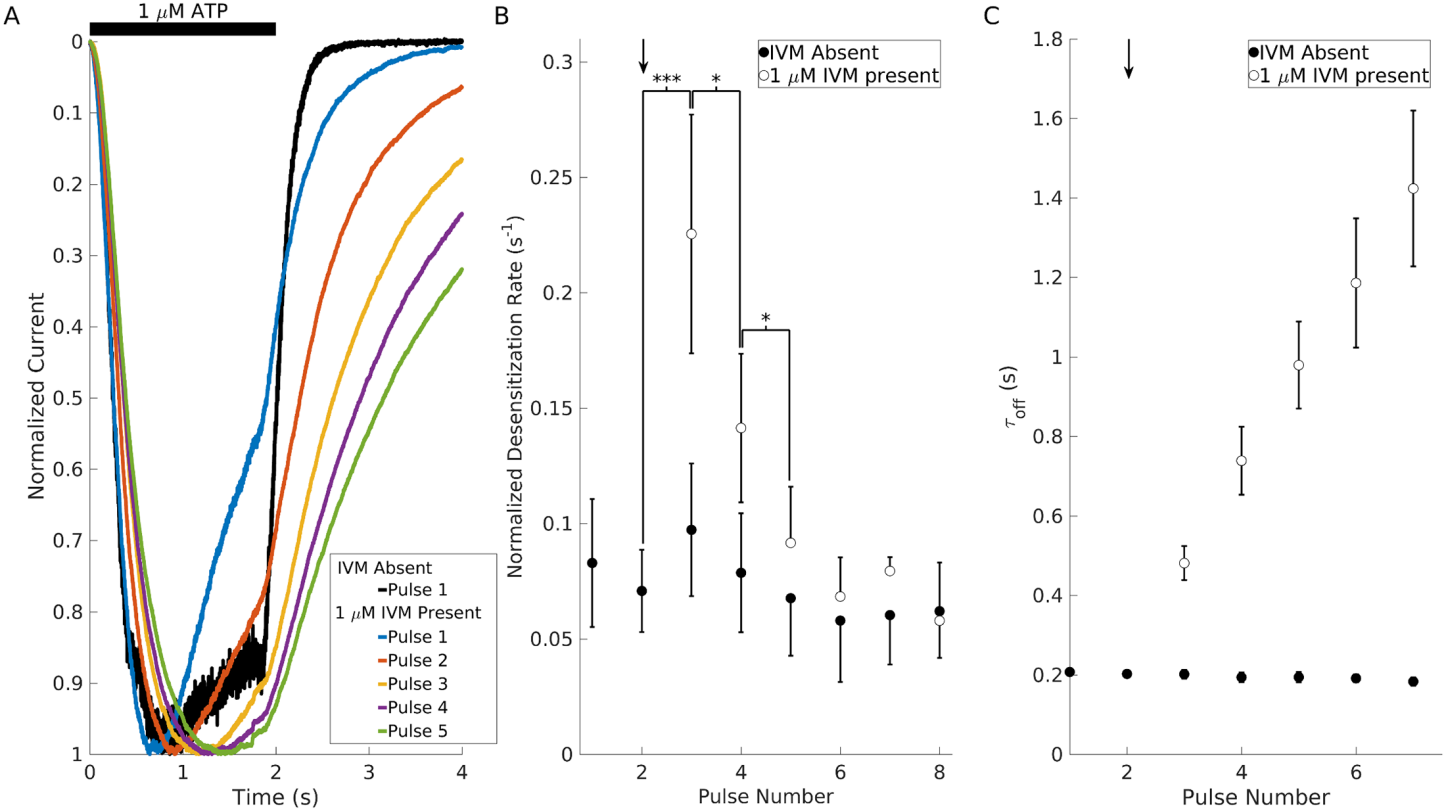
Effect of IVM on desensitization and deactivation kinetics in rat P2X4R. (A) Superimposed current pulses induced by 1 μM ATP and normalized by their maximum amplitudes, highlighting receptor kinetics during the pulse protocol (ATP application is highlighted by the black bar above the traces) performed with 1 μM IVM (derived from [20]). (B) Desensitization rates determined by linear fitting of the desensitization phase of each current pulse. In the absence of IVM (full circles), there was no significant variation in the desensitization rate, but following 1 μM IVM application (arrow), the desensitization rate significantly increased, followed by a gradual decline back to its value prior to stimulation with IVM. Statistical significance was calculated with the one sided Wilcoxon signed rank test. *p<0.05; ***p<0.005. (C) In the absence of IVM (full circles), deactivation kinetics remained constant after repeated agonist applications. In the presence of IVM (open circles), deactivation kinetics progressively slowed down with time. In A and B, data shown are means ± SEM from *n* = 7 records.

We next consider the effects of IVM on desensitization and deactivation. During the pulse protocol (Fig 1A), where cells were repeatedly stimulated by 1 μM ATP for 2 s twice per min in the absence (black trace) and presence of 1 μM IVM (colored traces), we observed an initial increase in desensitization rate of the receptor (blue trace in Fig 1A), followed by a gradual decrease in desensitization rate at each subsequent ATP pulse (see the Methods section for quantification procedure). By the fifth pulse (green trace in Fig 1A), the desensitization rate reverted back to a value comparable to that seen in the absence of IVM (black trace in Fig 1A).

To assess if this phenomenon occurs consistently, we evaluated the statistical significance of the transient increase in desensitization rate. To quantify the amount of desensitization seen in the recordings, we used linear fitting to measure the rate of receptor desensitization normalized by the current amplitude of each pulse. As shown in Fig 1B, we did not see a significant change in the rate of desensitization at each ATP pulse in the absence of IVM (filled circles) (n = 7), suggesting that the desensitization proccess of receptors is far from equilibrium. However, in the presence of IVM, the first two ATP pulses following IVM application (indicated by the small arrows) exhibited a significant (p < 0.005 and p < 0.05; n = 7) increase in desensitization rates. The desensitization rate of current recordings in subsequent ATP pulses gradually drifted back to its original value before IVM was applied, further suggesting that the open state, exhibiting an increased desensitization rate, has reached an equilibrium with its corresponding desensitized state. At higher IVM concentrations, however, these transient effects were not observed, but an increase in non-desensitized current amplitude was found [20]. Thus, while the binding of IVM potentiates P2X4R, it also increases both the apparent rate of desensitization at low ATP concentrations and the rate of recovery from desensitization (i.e., it lowers the Gibbs free energy barrier for these transitions).

To assess the deactivation kinetics (i.e., decay of current amplitude following washout of agonist) of P2X4R, we used the same pulse protocol of 1 μM ATP for 2 s twice per minute (Fig 1C). In the absence of IVM (filled circles), receptors underwent fast deactivation with a time constant that remained roughly the same at each pulse, whereas in the presence of 1 μM IVM (open circles following the small arrow) the deactivation time constant progressively increased with incubation time, indicating a decrease in receptor deactivation rates. This effect became even more pronounced at higher IVM concentrations. At IVM concentrations greater than or equal to 10 μM, deactivation following washout of IVM was not always complete (see Fig 2A in [20]), suggesting that complex physiological processes might be initiated at these concentrations. These results are consistent with the idea that IVM increases the sensitivity of the receptor to ATP and decreases the rate of agonist unbinding following its washout from medium [16–18].

### IVM causes receptor priming during ATP stimulation

Our previous study showed concentration response curves for rat P2X4R stimulated by ATP in the presence and absence of 3 μM IVM (S3 Fig); ATP alone was found to produce a concentration response curve with an EC_50_ of 2.3±0.4 μM (blue line), and with 30-s pretreatment with IVM, the EC_50_ was 0.5±0.1 μM (green line) [20]. A similar conclusion was reached with human P2X4R [17]. A pretreatment period of 10 s was also considered and, whereas it did produce the same maximal current amplitude, an intermediate EC_50_ of 1.6±0.3 μM was measured (maroon line) [20]. This suggests that there are at least two distinct priming effects associated with IVM with separate time scales of action. First, IVM primes receptors by increasing the maximal whole-cell current response. Second, after prolonged exposures to IVM, receptors become further primed by an increased sensitivity to ATP (previously called sensitization). The model in [20] was only partially able to account for these behaviors and specifically was not able to account for their dependence on the duration of pre-treatment because there were no kinetics associated with IVM binding in the absence of ATP.

### IVM increases open probabilities

The concentration response curves of P2X4R (S3 Fig) reveal that not only do 10- and 30-s pretreatments with IVM increase sensitivity to ATP (maroon and green lines, respectively), but they also increase the maximum current amplitude evoked by ATP [17, 20]. The two hypotheses that can explain this behavior are: (i) the unitary conductance of individual channels increases; or (ii) the number of open receptors is rising (i.e., the maximal open probability increases). Although there is evidence that the former hypothesis holds [17], this does not preclude a change as well in the maximal open probability with IVM application [17, 20, 23]. In fact, it was reported that the maximal open probability in the absence of IVM is ~0.2 compared to ~0.8 in the presence of IVM [17, 23]. This phenomenon was previously explained by the Markov model in [20], which assumes that IVM modifies the connectivity between open and desensitized states, but that model required a large increase in unitary conductance. This assumption on the conductance is inconsistent with a study of human P2X4R [17], which showed that IVM produces a roughly 5-fold increase in maximal current amplitude while only inducing a 20% increase in unitary conductance. Those authors posited that, in the absence of IVM, desensitization plays a large role in reducing the current amplitude, whereas when IVM is applied, desensitization is greatly reduced, enhancing the observed current. While this is a plausible explanation, we are not aware of any receptors that function in this manner. Moreover, no quantitative analysis was made to assess to what extent such a mechanism produces the observed effect.

**Fig 2:**
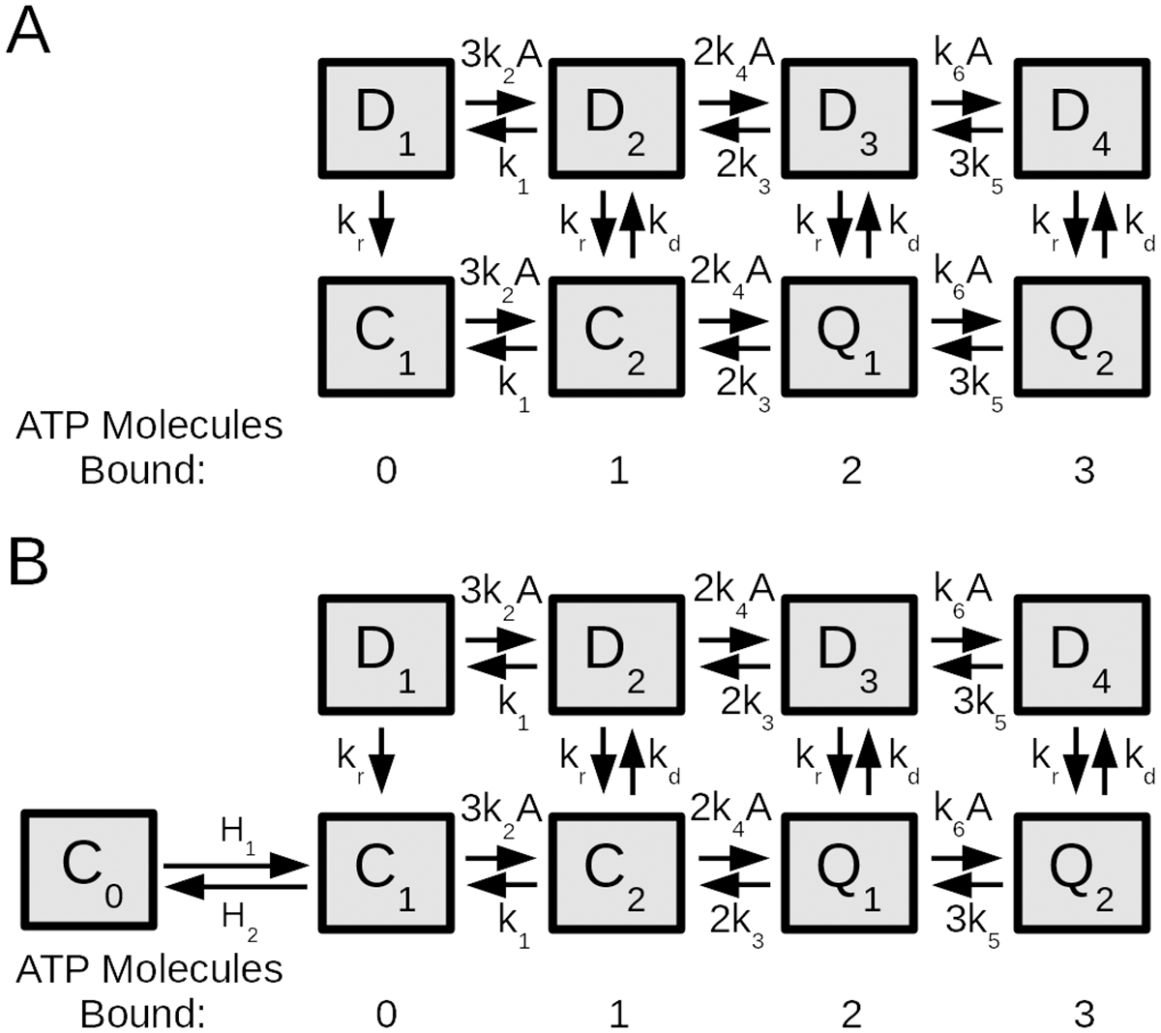
Generic desensitization schemes of ligand-gated ion channels with three agonist binding sites. (A) A diagram of the underlying orthosteric (ATP) gating scheme based on previously developed P2XR models, consisting of closed (*C*_1_, *C*_2_), open (*Q*_1_, *Q*_2_) and desensitized (*D*_1_, *D*_2,_ *D*_3_, *D*_4_) states, each representing the fraction of receptors at a given state. The forward (backward) rates of agonist binding (unbiding) are represented by *k*_2_, *k*_4_, *k*_6_ (*k*_1_, *k*_3_, *k*_5_), where *A* denotes the concentration of agonist, and the desensitization (recovery) rates are denoted *k*_*d*_ (*k*_*r*_). (B) A diagram of the gating scheme in A augmented by a deeply inactivated state *C*_0_ and two additional transition rates *H*_1_, *H*_2_.

In order to test this hypothesis, we constructed a simple and generic mathematical scheme (hereafter referred to as a gating scheme) of a desensitizing ligand-activated receptor (Fig 2A). It consists of two rows: a naïve row comprised of two closed states (*C*_1_, *C*_2_) and two conducting states (*Q*_1_, *Q*_2_), and a desensitized row comprised of four nonconducting desensitized states (*D*_1_, *D*_2_, *D*_3_, *D*_4_). As was done in [20], we assume that channels open from states with two or three bound ATP molecules. This is in accordance with the finding that a single-bound receptor state does not lead to activation of P2X7 channels [28]. This is also consistent with previous models of P2XRs and the notion that a single kinetic model underlies the functioning of all receptor subtypes [19, 20, 24–26, 29]. Forward (backward) transitions between two states along each row represent a single ATP binding (unbinding) with rates *k*_2_, *k*_4_, *k*_6_ (*k*_1_, *k*_3_, *k*_5_), respectively, whereas upward (downward) transitions represent desensitization (recovery) with a rate *k*_*d*_ (*k*_*r*_). Concentration response curves were generated for this gating scheme, each with a progressively increasing rate of desensitization *k*_*d*_ (see Figs 2A and S5A). It was found that although reduced desensitization rates are capable of increasing the current amplitude at a given agonist (such as ATP for P2X4R) concentration, the mechanism proposed in [17] is unable to significantly increase the maximal current amplitude evoked by the agonist. Rather, it shifts the EC_50_ of the concentration response curves leftward as well as increases the Hill coefficient in such a way that the saturating phase of the concentration response curves are shifted by many orders of magnitude. A leftward shift in EC_50_ and modulation of the Hill coefficient by IVM have been observed experimentally [17, 20]. This was, however, consistently associated with an increase in the maximal current amplitude, which the desensitization mechanism cannot produce at saturating agonist concentrations (see I_max_ in the legend of S5A Fig). A mathematical model introduced by Silberberg et al. also used an IVM-dependent transition rather than modulation of desensitization by IVM in order to produce the increase in maximal current [23]. Therefore, the mechanism suggested in [17] seems unable, at least on its own, to explain the effects of IVM on the concentration-response relationship for the peak current of P2X4R.

After having ruled out decreased desensitization as a cause for the increased maximal current amplitude in the presence of a modulator, we tested an alternative hypothesis, that the closed states exist in equilibrium with a deeply inactivated state (*C*_0_) for which the agonist is not effective (Fig 2B). This mechanism has previously been used in Markov models of sodium channels [30]. Transitions linking the two states must be slow, but the equilibrium mixture of the closed-inactivated subsystem (*C*_0_ ↔ *C*_1_) establishes an upper bound on the maximal open probability in the absence of IVM, given by

$$P_{O}^{Max}=\frac{H_{1}}{H_{1}+H_{2}}$$ (6)

even at the highest agonist concentration. In other words, before agonist application, only some fraction of receptors are in *C*_1_ and are susceptible to agonist-induced activation but in the presence of IVM, more can be recruited (from *C*_0_) into *C*_1_. Evidence of such recruitment was first seen during prolonged application of ATP in the absence and presence of IVM [20] and was obtained also by application of IVM to fully desensitized receptors (S4 Fig). To see how effective this mechanism is in producing the observed effects in [17], we tested the gating scheme of Fig 2B quantitatively, by progressively increasing the transition rate *H*_1_ (between *C*_0_ and *C*_1_), and plotting the concentration response curves (S5B Fig). Increasing *H*_1_ decreased inactivation, which increased the fraction of receptors in *C*_1_ and thus $P_{O}^{Max}$.

Whereas reducing the occupancy of the deeply inactivated state is highly effective at increasing the maximal current amplitude, it does not significantly shift the concentration response curves or alter the Hill coefficients. Therefore, in order to match the experimental findings that IVM pretreatment of P2X4R not only increased maximal current but also shifted the EC_50_ leftward (S3 Fig) and increased the Hill coefficients, both reduced desensitization and rescue from a deeply inactivated state seem to be required. A model incorporating both features is described in the next section.

### One-layer model

Based on the above considerations, we designed a one-layer Markov state model that describes the full kinetics of ATP and IVM binding to P2X4R and tested it against experimental data. For a detailed description of the model, see S6 Fig, Table A and Appendix A in S1 Text. Briefly, it is a revised version of the model of Zemkova et. al. [20] that now assumes 3 IVM binding sites, that the binding of IVM acts on P2X4R independently of ATP binding, and that IVM can bind to any ATP-bound state, not just the 3-ATP bound naïve state. Sequential binding of IVM causes three stages of receptor priming, depending on number of IVM molecules bound to receptor: primed-1, primed-2, and primed-3. Primed-1 receptors respond to ATP application with increased current amplitude, reflecting increased open probability. Primed-2 receptors exhibit modestly increased unitary conductance for Na^+^ and significantly increased unitary conductance for NMDG^+^, whereas primed-3 receptors show increased ATP binding affinity. The model also incorporates rescue from the deeply inactivated state by IVM, and therefore has a maximal open probability given by Eq (6) in the absence of IVM.

Although our analysis of this model (and several variations of it) revealed that it possesses many of the necessary ingredients to capture the gating properties of P2X4R and several aspects of its current recordings (S7 and S8 Figs), it includes the implausible assumption that receptors in the primed states must lose all bound IVM molecules in order to desensitize. This assumption led to two major issues in the performance of the model: (i) it did not capture accurately the short timescales of activation and desensitization robustly; and (ii) it produced discrepancies in current amplitudes when compared to experimental data during the pulse protocol. That motivated us to design a more accurate model of P2X4R kinetics.

### Two-layer model

#### Receptor stages and gating schemes

The observed transient increase in desensitization of P2X4R immediately after IVM application, followed by a gradual decrease in this rate (Fig 1), suggests that IVM bound states of the receptors are also directly associated with desensitization. This type of behaviour cannot be reproduced well by Markov models lacking this feature (such as the model in [20] or the one-layer model). Because it was more reasonable to suggest that IVM modifies receptor desensitization rather than preventing it from happening altogether, we investigated the effect of allowing reversible transitions between IVM-bound states and their corresponding desensitized states. This was done by including, in addition to the IVM-induced modified ATP kinetics (which produce the slowed deactivation rates and increased sensitivity to ATP), another IVM bound row with modified desensitization kinetics that can readily recover from desensitized states. According to both the simple gating schemes of Fig 2 (taken from [20]) and the one-layer model (S6 Fig), the submodel formed by removing the IVM-bound states allows receptors to return from the desensitized row with a rate that is independent of IVM-related processes. We hypothesized that the gating scheme in Fig 2A is the underlying system whose rate parameters are modified with each IVM binding to produce the varied kinetics we observe at different IVM and ATP concentrations.

To test this, we chose two recordings of the pulse protocol performed at 1 μM and 10 μM IVM (see the experimental recordings in S9 Fig). Choosing the first pulse (before IVM application) and the last pulse (before IVM washout) from each recording, respectively, we were able to fit the transition rate parameters of the gating scheme in Fig 2A to these pulses very accurately (see S9A and S9C Fig). In particular, this model captured the fast activation, desensitization, and insensitivity to ATP removal at 10 μM IVM with one set of parameters for each case. However, S9B Fig shows that although the gating scheme reproduced the first pulse extremely well, by the last pulse, both the deactivation kinetics and current amplitude were off. Similarly, S9D Fig shows good agreement between the last pulse and simulation while the first few pulses of the recording were off. These results suggest that a mixture of these gating schemes must be linked by IVM dependent transition rates, and that (i) at the lowest IVM concentrations, the mixture will be predominantly composed of naïve receptors and receptors which have only undergone a single modification by IVM, and (ii) at the highest IVM concentrations, the mixture will eventually saturate at these states that are most modified by IVM. In other words, we expect that building a model in which gating schemes of the type depicted in Fig 2A are allowed to mix, will better capture the complex experimental behaviour.

#### Description of the two-layer model

Motivated by the fact that the same underlying gating scheme could be used to reproduce single pulses of the pulse protocol with great fidelity at all IVM concentrations, we set out to develop a model which could capture not only the short timescale behaviour of receptors but also the progression in kinetics and amplitude which the single gating scheme failed to capture. When cooperativity between IVM and ATP was included in simple models (such as the one-layer model), there was a clear progression in kinetics and amplitude. With this in mind, we have investigated a new model that included a mixture of gating schemes shown in Fig 2A and 2B. Because we allow for all closed and open states to desensitize, we termed this model the two-layer model.

**Fig 3:**
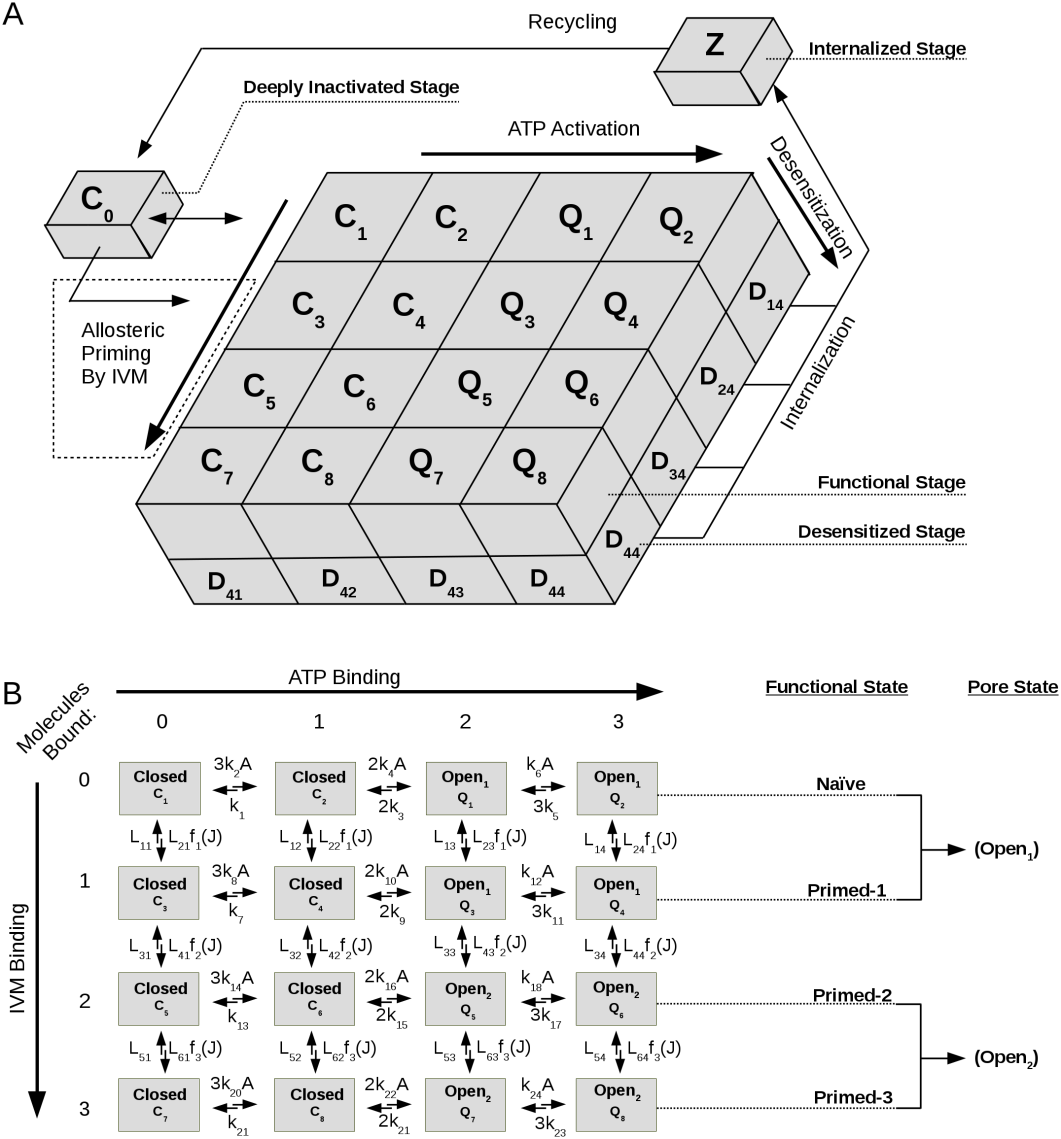
Diagram of the two-layer Markov state model describing the sequential binding and unbinding of ATP (denoted by *A*) and IVM (denoted by *J*) to P2X4R. (A) The activation cycle of P2X4R channels. In the two-layer model, receptors exist in four different stages: functional, desensitized, internalized, and deeply inactivated. The upper layer represents the functional stage of receptors, with closed, *C*_*i*_, and conducting, *Q*_*i*_, states, *i* = 1, 2, ⋯, 8, and a lower layer represents the nonconducting desensitized stage comprised of *D*_*jk*_ states, *j*, *k* = 1, 2, 3, 4. Two additional stages, *Z* and *C*_0_, represents internalized and deeply inactivated states, respectively. (B) The occupancy of orthosteric and allosteric binding sites and the pore states of receptors in functional stage. Transitions between states along rows represent binding (rightward) or unbinding (leftward) of one ATP, and along columns represent binding (downward) or unbinding (upward) of one IVM. Receptors are in naïve or primed functional states, and receptors are primed with 1, 2 or 3 molecules of IVM. States *Q*_1_,*Q*_2_,*Q*_3_ and *Q*_4_ are open with maximum conductance *g*_1_ and reversal potential *E*_1_ (open_1_) whereas *Q*_5_,*Q*_6_,*Q*_7_ and *Q*_8_ are open with maximum conductance *g*_2_ > *g*_1_ and reversal potential *E*_2_ < *E*_1_ (open_2_). Before stimulation with ATP and IVM, receptors reside in *C*_0_ or *C*_1_. Parameter values are listed in Table C in S1 Text.

Fig 3A describes the two-layer model in detail. P2X4Rs are assumed to be found in four stages during their activation cycle: deeply inactivated, functional, desensitized, and internalized. The upper layer consists of closed (*C*_1_−*C*_8_) and conducting (*Q*_1_−*Q*_8_) states, which we termed functional states. Fig 3B shows how the functional states are determined by binding of ATP and IVM, producing four subgroups of functional states: naïve, primed-1, primed-2, and primed-3. Naïve and primed-1 channels, which differ in their maximum probability of opening, respond to binding of 2 or 3 ATP molecules with open_1_ states. Primed-2 and primed-3 channels respond to ATP with open_2_ (NMDG^+^-conducting) states, but primed-3 receptors also exhibit increased sensitivity to ATP. The model was constructed by linking 4 distinct gating schemes together (each consisting of 4 functional states and 4 desensitized states) through fourth-order Hill functions that depend on IVM concentrations (see Appendix B and Table C in S1 Text). Their lower layer is composed of nonconducting desensitized states. This effectively means that desensitization is a process which does not modify agonist binding but rather affects the receptor-channel by making it nonconducting despite agonist binding. Furthermore, we allowed for internalization from all desensitized states which have three bound ATP molecules. The rate of internalization from the naïve states (*H*_3_) was assumed to be different from that of IVM bound states (*H*_5_) to reflect that IVM alters endocytosis of P2X4R receptors [31].

The results of Fig 1 imply that IVM not only modifies ATP-binding and activation kinetics, but also shifts the receptor population into states where the transition to desensitized states is less favorable. To obtain such behaviour in the model, we must ensure that the ratio of desensitization-to-recovery rates associated with the naïve states is much larger than those of the primed states, i.e., the following condition must be satisfied:

$$\frac{k_{d,1}}{k_{r,1}}\gg \left\{\frac{k_{d,2}}{k_{r,2}},\frac{k_{d,3}}{k_{r,3}},\frac{k_{d,4}}{k_{r,4}}\right\}.$$

#### Fitting of the two-layer model to the pulse/prolonged protocols

**Fig 4:**
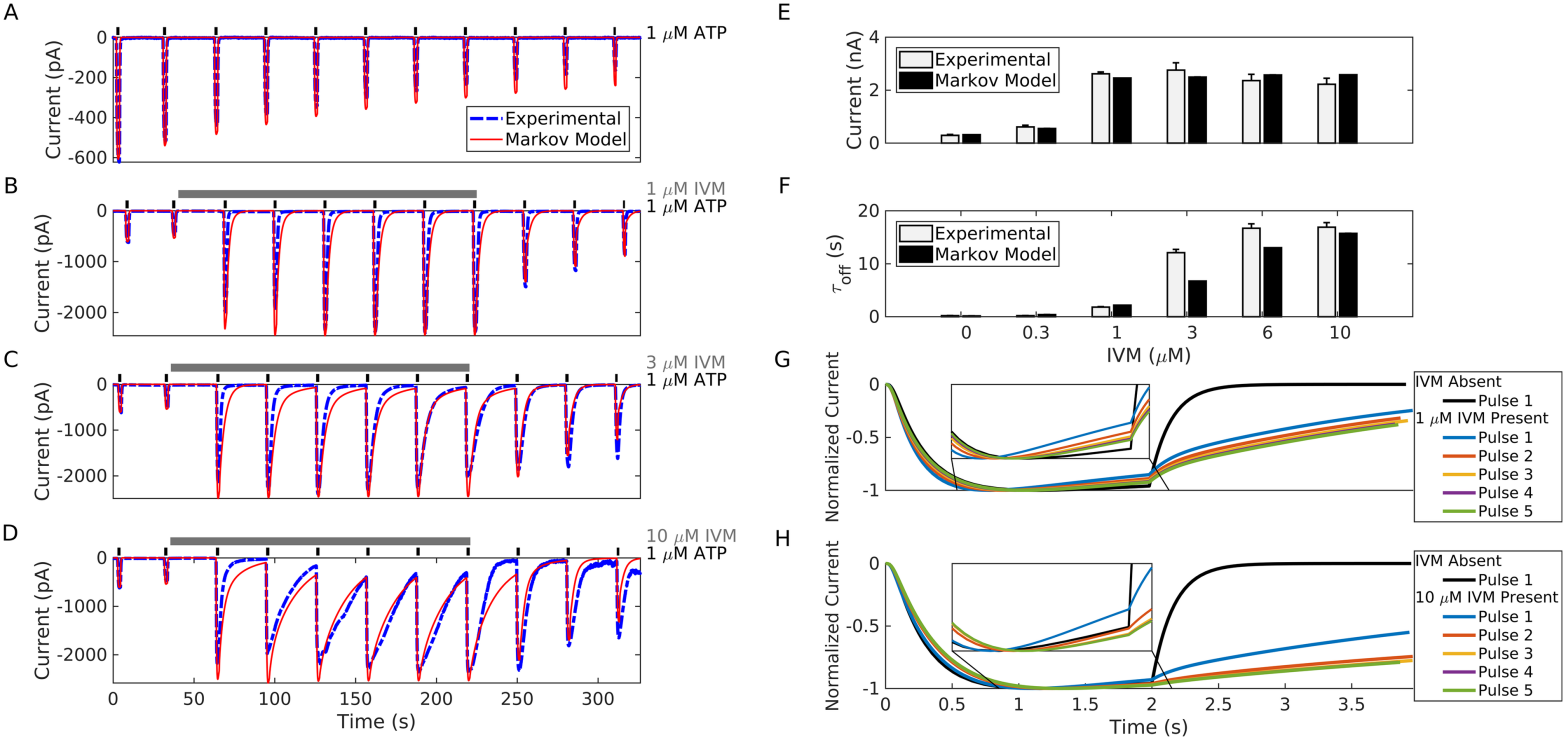
Outcomes of the two-layer model determined by the MCMC fitting to the pulse and prolonged protocols. (A-D) Simulated time series of current for the pulse protocol performed with (A) 0, (B) 1, (C) 3, and (D) 10 μM IVM. Dashed blue lines, experimental recordings; solid red lines, simulations. (E, F) IVM-dependent concentration-response changes in (E) peak current amplitude and (F) rate of receptor deactivation. Model deactivation kinetics *τ*_*off*_ measured by the weighted time constant. (G, H) Progression of activation, desensitization, and deactivation of currents produced by the model and normalized by maximum amplitudes during the pulse protocol in the presence of 1 μM (G) and 10 μM (H) IVM. Insets show the magnified desensitization phases of the response. All experimental data are derived from [20].

**Fig 5:**
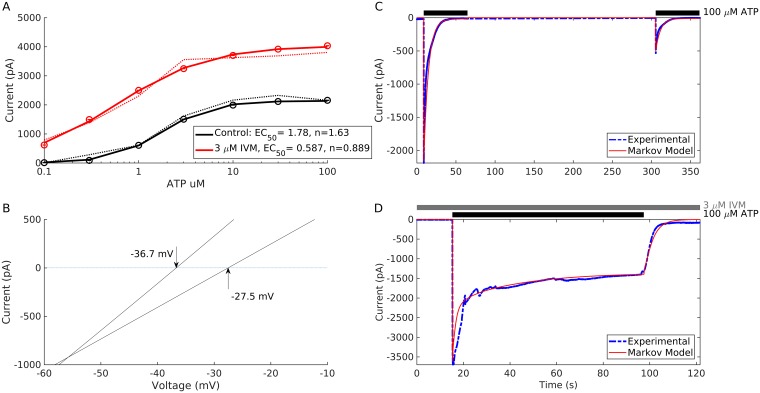
Outcomes of the two-layer model determined by the MCMC fitting to the pulse and prolonged protocols. (A) ATP-dependent concentration-response curves in the absence (black) and presence (red) of 3 μM IVM applied 30 s before ATP stimulation. Calculated EC_50_ and Hill coefficients (n) in the absence of IVM were 1.78 μM and 1.63, respectively, and in the presence of IVM were 0.587 μM and 0.889, respectively. Dotted lines are experimental data. (B) The first and last I-V curves showing the decrease in their slopes and a positive shift in reversal potential upon stimulation with 100 μM ATP for 10 s in the presence of 3 μM IVM applied 20 s before ATP stimulation. The bath medium had Na^+^ replaced by NMDG^+^ modeled by setting *E*_1_ = −93.5 mV and *E*_2_ = −5.1 mV. Voltage was ramped from − 80 mV to + 80 mV twice per second from a holding potential of − 60 mV. (C) Two prolonged applications of 100 μM ATP separated by a 4 min washout period in the absence of IVM. (D) Prolonged application of 100 μM ATP produced by the model in the presence of 3 μM IVM. Dashed blue lines, experimental recordings; solid red lines, simulations. All experimental data were derived from [20].

In order to assess the capacity of the model to reproduce P2X4R gating, we used MCMC techniques (see Methods Section) to determine the parameter values that reproduce the kinetics observed experimentally during the pulse and prolonged protocols. The results shown in Figs 4 and 5 reveal that the model is able to capture every aspect of P2X4R gating, including naïve receptor activation, desensitization, and deactivation (Fig 4A, 4E and 4F) in the absence of IVM, and the increase in current amplitude (Fig 4B–4E) and deactivation time constant (Fig 4F) upon IVM application during the pulse protocol (compare experimental recordings in blue curves/white bars to simulations in red curves/black bars). The transient increase in desensitization rate after 1 and 10 μM IVM application followed by a progressive decrease in desensitization rate at each subsequent pulse (Fig 4G and 4H), along with the insensitivity of P2X4R to ATP removal during washout (Fig 4H) in the pulse protocol were also captured by the model. The ATP-dependent concentration response curves for current amplitude generated by the model during the pulse protocol in the absence (black) and presence (red) of IVM applied 30 s before ATP stimulation (Fig 5A) also exhibited a leftward shift (from ~1.78 μM to ~0.587 μM) in the EC_50_ in a manner almost identical to that seen experimentally (dotted lines). These results suggest that the model can reproduce the kinetics of the receptors occurring at short time scales, that having desensitization decoupled from IVM unbinding allowed the model to produce the transient increase in desensitization during low IVM stimulation (i.e., in primed-1 cells) and that (pre)stimulation with moderate to high IVM concentrations rescues receptors from desensitization (through an increase in the rate of return from desensitization) within this time scale.

Comparing the observed data during the ramp protocol to the outcomes of the model shows that the model can reproduce the decrease in the slope of the I-V curves as demonstrated by the first and last I-V curves generated by applying a voltage ramp from − 80 mV to + 80 mV during 10 s of 100 μM ATP stimulation in the presence of 3 μM IVM applied 20 s prior to ATP stimulation (Fig 5B). Here we focused on the first and last I-V curves because we are not tracking ionic fluxes within the model (see Appendix C in S1 Text). This phenomenon was robustly produced by ensuring that Inequality Eq (5) was satisfied by model simulations. The current equations (Eq (S1)) can also produce a shift in reversal potential from −36.7 mV to − 27.5 mV, similar to that observed experimentally in the presence IVM under bionic (NMDG^+^ out/ Na^+^ in) conditions. Based on the Goldman-Hodgkin-Katz equation, this was achieved by generating a value for *E*_1_, the reversal potential of the open state (open_1_), using the MCMC algorithm, that is more negative than that of the primed state *E*_2_ (see Appendix C in S1 Text). It is important to note that (i) this shift in reversal potential can be still generated in the absence of an increase in unitary conductance, when *g*_1_ = *g*_2_, as shown in S12B Fig, indicating that the shift is independent of an increase in unitary conductance; and (ii) we do not explicitly model the flux of cations through the pore, and therefore the reversal potential *E*_2_ reflects both the altered selectivity of the open_2_ pore and the accumulation of NMDG^+^ within the cell (as was demonstrated in [13]).

Finally, comparing the experimental recordings (blue) to model simulations (red), shows that the two-layer model is successful in reproducing the responses obtained in prolonged application of 100 μM of ATP in both the absence (Fig 5C) and presence of 3 μM IVM (Fig 5D). The former showed partial recovery of peak current response after 4-min ATP washout, whereas the latter showed a significant increase in amplitude and a plateauing in current amplitude towards the end of the desensitization phase, as seen experimentally. These results suggest that a substantial fraction of P2X4R receptors are shielded from desensitization in the presence of IVM during prolonged time scales (by transitioning receptors to the primed gating schemes) and that these four processes exist at equilibrium during the plateauing phase.

#### Kinetics of the two-layer model

The structure of the two-layer model and the fitted parameter values (Table C and Appendix B in S1 Text) suggest that agonist potency is increased by IVM which rescues receptor population from a pool of inactivated receptors (*C*_0_). The gating scheme of the primed-1 subsystem (i.e., with one IVM bound) had an increased desensitization rate which led to the observed transient increase in desensitization after IVM application and was also found to exhibit negative cooperativity (see Methods). The increased sensitivity to ATP in this and other IVM-bound schemes was produced by having their ATP unbinding rates decrease and/or their ATP binding kinetics increase. The model also revealed that the recovery rates of all the IVM-bound gating schemes are significantly larger than that of the naïve gating scheme (i.e., *k*_*r*,*i*_ ≫ *k*_*r*,1_, *i* = 2, 3, 4) while the internalization rate of these schemes is lower than that of naïve receptors (i.e., *H*_5_ < *H*_3_), which leads to the long-lasting currents observed during prolonged ATP applications in the presence of IVM (Fig 5D; for additional current recordings with the same protocol, see S10 Fig). Finally, the unitary conductance increase estimated by MCMC was approximately 15%, which is within the experimental bound. Together, these results show that, in this model of P2X4R, unitary conductance increase is present but plays a minor role in increasing the current amplitude in the presence of IVM, whereas the open probability is the key to producing such large currents.

## Discussion

Data-based Markov state models that describe the processes of ligand binding/unbinding to ligand-gated receptor are powerful tools to understand orthosteric and allosteric regulation of these channels. P2X4Rs are prototypical examples of such receptors with orthosteric and allosteric binding sites for ATP and IVM, respectively. They are associated with ion channels that are permeable to small cations, including Na^+^, Ca^2+^, Mg^2+^ and K^+^. The binding of ATP leads to receptor activation and channel opening, while IVM binding increases receptor unitary conductance and sensitivity to ATP. Here we analysed the kinetics of ATP and IVM binding/unbinding to P2X4R, and determined its gating properties using two detailed Markov models labelled the one-layer and two-layer models.

The one-layer model extended a previously developed, simple Markov model of P2X4R by taking into consideration a deeply inactivated state, nonresponsive to ATP but responsive to IVM (existing in equilibrium with the naïve ATP-unbound state), along with three additional gating schemes (per each ATP binding) representing the three IVM binding sites. The model also assumed that the IVM and ATP binding are independent of one another and that sequential binding of IVM can occur at any ATP-bound or unbound states. Our analysis revealed that the deeply inactivated state was essential for capturing the increase in the maximum response (I_max_) in the ATP-dependent concentration-response curves (in the presence of IVM), with only a small increase in conductance between the open_1_ and open_2_ states. Although the model was able to capture many of the essential features of P2X4R recordings (S7 and S8 Figs), it assumed that IVM bound states can only desensitize by first becoming completely free of IVM. That made the model unable to robustly capture the short timescales of activation and desensitization, and it also produced current amplitudes incompatible with experimental data during the pulse protocol.

By allowing the IVM-bound states to desensitize, we were able to show that a simple gating scheme is able to capture the profiles of the first pulse (in the absence of IVM) or the last pulse (in the presence of IVM) of the pulse protocol very accurately when the scheme is fitted individually to each pulse, but not both simultaneously. However, when comparing the entire pulse protocol recording to the outcome of the scheme for each case, there was a gradual increase in discrepancy between them, suggesting that a mixture of gating schemes must coexist to be able to capture all aspects of P2X4R kinetics. That led us to propose the two-layer model, which assumes that IVM-bound states can desensitize.

The two-layer model was successful in capturing every aspect of P2X4R kinetics very accurately, including the short and long time scales of activation and desensitization, particularly the changes in the desensitization rate observed during the pulse protocol of Fig 1, as well as the current amplitudes. The observed shift in the EC_50_ along with the increase in the maximum current, during pre-stimulation with IVM, were also reproduced by the model (through the presence of the deeply inactivated state). Moreover, these gating schemes can be used to understand why ATP binding mutants with low amplitude of response tend to have significantly larger fold-increases in maximal current in the presence of IVM [21]. If we view such mutants as disproportionately populating the deeply inactivated state, where they cannot bind ATP, then their rescue by IVM from this state will produce a much larger fold-increase in maximal current. The existence of this deeply inactivated state was probed experimentally, by applying IVM to a cell whose receptors were almost completely desensitized from prolonged applications of 100 μM ATP. Upon IVM application, we observed an increase in the maximal current amplitude to about half of the initial maximal current (see S4 Fig), suggesting that IVM rescued receptors from a (deeply inactivated) pool corresponding to about one third of all receptors.

The two-layer model consisted of 4 gating schemes linked together through ATP/IVM binding/unbinding. Two of these gating schemes (the primed-2 and -3) contained conducting states exhibiting a 15% increase in unitary conductance compared to that of the open states in the naïve and primed-1 states. This increase is within the 20% limit seen experimentally [17], and is not required to produce IVM’s increase in maximal current amplitude within our model. Instead, the effect of IVM on maximal current amplitude is produced mainly by an increase in open probability. According to Eq (6), the two layer model predicts a maximal open probability of approximately 0.53 in the absence of IVM (with 47% of receptors in a deeply inactivated state), while it can easily reach values greater than 0.9 in the presence of IVM. It was suggested in [13] that the ionic conditions in the medium and the pipete are responsible for producing electrochemical effects which were long presumed to be evidence for pore dilation, particularly in P2X2R. While temporal changes in ionic gradients play a significant role in producing a shift in E_rev_ associated with the I-V curves during the ramp protocol, our results suggest that the transition to open_2_ (which is permeable to NMDG^+^) is an intrinsic property of the pore in P2X4R, is independent of the increase in unitary conductance (S12B Fig) and is induced by priming with IVM. The two-layer model assumes that the increase in unitary conductance associated with this transition is masked by desensitization. This results in the shift in E_rev_ being accompanied by a decrease in the slope of the I-V curves (due to desensitization). The observed decline in the slope of the I-V curves was a consequence of Inequalities Eqs (3) and (5). The previously developed model in [20] was capable of fulfilling these conditions and producing the decrease in the slope of the I-V curves, but it required a large increase (>150%) in unitary conductance to achieve it while simultaneously producing the increase in current amplitude induced by IVM. The inclusion of the primed-1 row with conductance *g*_1_ (and reversal potential *E*_1_) was an essential element for reproducing the shift in the I-V curves. Without this intermediate step, the model required a very positive reversal potential for the open_2_ state (*E*_2_), which does not reflect its loss of selectivity. Both the one-layer and two-layer models proposed here keep the increase in unitary conductance within 20% and produce the current growth with IVM pretreatment through IVM-induced transitions from the inactivated state *C*_0_ (by increasing $P_{O}^{Max}$, as given by Eq (6), rather than increasing the maximal conductance). The two-layer model, however, is more plausible because it does not assume that desensitization necessitates the unbinding of IVM. Moreover, according to this model, IVM is able to transition receptors to the primed-3 states in the absence of ATP, allowing pretreatment with IVM to produce sensitization independently of ATP. It is important to note that effectively removing the 15% increase in unitary conductance associated with the primed states only slightly altered model simulations and did not abolish any experimental phenomena in symmetric ionic conditions (S11 and S12 Figs). Thus, the results of the two-layer model are independent of an increase in unitary conductance, but require a change in selectivity in order to capture the shift in the I-V curves of the ramp protocol.

Recently, the pore dilation hypothesis has become increasingly disputed. Molecular dynamics simulations indicate that NDMG^+^ is capable of permeating the open state of P2X4R pore, provided it is maintained in an open state long enough for the slow permeation event of NDMG^+^ to take place [15, 32]. One of the primary effects of IVM application on the single channel kinetics of P2X4R is to shift the distribution of open times from the sub-millisecond timecale to tens of millisecond [17]. We hypothesize that the drastic change in P2X4R’s permeability for NMDG^+^ upon IVM application results from the priming of receptors in such a way that their pores remain in the open_2_ state for long enough for the slow permeation of NMDG^+^. This increase in the permeability for NMDG^+^ (via an IVM-dependent transition to the open_2_ state) allows for its influx into the cell. Together with the efflux of Na^+^, these fluxes produce a more positive E_rev_ as determined by the Goldman-Hodgkin-Katz equation.

While the two-layer model may seem to be a large departure from both the previously developed model in [20] and the one-layer model, it should be noted that in the absence of IVM, the remaining blocks of the models (or submodels) are identical, and that the P2X2R Markov model developed in [19] has a similar structure; it included a corresponding desensitized state for each of its closed and open states, although the desensitization pathway for primed (sensitized) states was calcium dependent. The increase in the number of states and number of kinetics parameters in the two-layer model was necessary to capture all the observed features of P2X4R, which previous models, including the one-layer model presented here, failed to do. A step by step validation of such an increase in complexity was provided through the use of coupled gating schemes, and the design of an extensive MCMC fitting algorithm that combined parallel tempering approaches with the t-walk method to estimate the kinetic parameters of the model efficiently.

The two major allosteric effects of IVM’s on P2X4Rs, observable from whole cell currents as an increase in maximal current amplitude or the deactivation time constant, exhibit distinct concentration dependencies [17, 20]. This suggests that they are likely caused by two independent processes. This existence of two distinct allosteric effects with differing concentration dependencies have also been reported for other P2XRs [11, 19], although for these receptors, ATP alone was sufficient to induce such effects. The models presented here reduce the concentration dependence of IVM’s allosteric effects on P2X4R to a single sequential binding process, and thus represent a major simplification of a more realistic model where all effects are assumed to arise from independent binding events. Despite this simplification, the model is quite capable of capturing all aspects of allosteric modification by IVM.

An important item for future work is cooperativity in the ATP and IVM binding, which has been investigated in the one-layer model but not yet in the two-layer model. By assuming correlations between the binding/unbinding parameters of ATP and IVM in the one-layer model, we were able to reduce the number of estimated parameters in that model significantly and found that there was negative cooperativity in the ATP binding in the naïve and primed-1 rows. Investigating if such cooperativity exists in the two-layer model is also warranted. This can be done by imposing correlations between the kinetic parameters of the two-layer model, which will again reduce the number of estimated parameters, and testing for cooperativity in ATP binding, IVM binding and between ATP and IVM binding. These variations of the model can then be compared to each other using Bayesian approaches to determine which is most likely.

In conclusion, here we present two novel models, one of which (the two-layer model) effectively mimics all experimental observations. In this model, receptors go through four stages of activation cycle during ATP and IVM binding: transitioning from functional to desensitized, from desensitized to internalized, from internalized to deeply inactivated and from deeply inactivated to functional. Functional and desensitized stages each exist as 16 distinct states, determined by the progressive saturation of three ATP and three IVM binding sites, whereas internalized and deeply inactivated receptors are single states. Binding of IVM influences ATP-induced gating properties of receptors, i.e. the rates of activation, desensitization and deactivation, open probability of channels, and the sensitivity of receptors to ATP. The channel pore state, open_1_ is predominantly permeable to small cations and open_2_ is permeable to large organic cations.

## Methods

### Cell cultures and transfection

Experiments were performed on human embryonic kidney 293 cells (HEK293; American Type Culture Collection), which were grown in Dulbecco’s modified Eagle’s medium supplemented with 10% fetal bovine serum, 50 U/mL penicillin, and 50 μg/mL streptomycin in a humidified 5% CO_2_ atmosphere at 37°C. Cells were cultured in 75-cm^2^ plastic culture flasks (NUNC, Rochester, NY, USA) for 36–72 h until they reached 80–95% confluence. Before the day of transfection, ~150 000 cells were plated on 35 mm culture dishes (Sarstedt, Newton, NC, USA) and incubated at 37°C for at least 24 h. For each culture dish of HEK293 cells, transfection of wild-type P2X4R was conducted using 2 μg of DNA with 2 μl of jetPRIME reagent in 2 ml of Dulbecco modified Eagle’s medium, according to the manufacturer’s instructions (PolyPlus-transfection, Illkirch, France). After 24–48 h of incubation, the transfected cells were mechanically dispersed and re-cultured on 35 mm dishes of Corning 3294 CellBIND Surface for 1–4 hours before recording. Transfected cells were identified by the fluorescence signal of enhanced green fluorescent protein using an inverted research microscope with fluorescence illuminators (Model IX71; Olympus, Melville, NY).

### Patch-clamp recordings

Currents were recorded in a whole-cell configuration from cells clamped to −60 mV using an Axopatch 200B patch clamp amplifier (Axon Instruments, Union City, CA, USA). All currents were captured and stored using Digidata 1550A and pClamp10 software package. Patch electrodes were pulled from borosilicate glass tube with a 1.65 mm outer diameter (type GB150F-8P; Science Products GmbG, Hofheim, Germany) using a Flaming Brown horizontal puller (P-97; Sutter Instruments, Novato, CA). The tip of the pipette was heat-polished to a final tip resistance of 3–5 MOhm. During the experiments, the dishes with cell cultures were perfused with an extracellular solution containing: 142 mM NaCl, 3 mM KCl, 2 mM CaCl_2_, 1 mM MgCl_2_, 10 mM HEPES and 10 mM D-glucose, adjusted to pH 7.3 with 10 M NaOH. The osmolarity of solution was 290 mOsm as determined by a vapor pressure osmometer (Model VAPRO 5520; Wescor, Logan, UT, USA). Experiments were done on single cells with an average capacitance of about 10 pF held at membrane potential of -60 mV. Patch electrodes used for whole-cell recording were filled with an intracellular solution containing: 145 mM NaCl, 10 mM EGTA and 10 mM HEPES; the pH was adjusted with 10 M NaOH to 7.2. The osmolality of the intracellular solution was 293 mOsM. Current-voltage relations were obtained by voltage ramps from −80 mV to +80 mV twice per second and used to estimate changes in reversal potential during 10–30 s of agonist application (Yan et al., 2008). Under a ramp protocol, cells were bathed in extracellular solution containing: 155 mM *N*-Methyl-d-glucamine (NMDG^+^), 3 mM KCl, 2 mM CaCl_2_, 1 mM MgCl_2_, 10 mM HEPES and 10 mM D-glucose, adjusted to pH 7.3 with HCl, if not otherwise stated. IVM was dissolved in dimethyl sulfoxide, stored in stock solutions at 10 mM, and diluted to required concentration in extracellular solution prior to experiments. The control, ATP-containing and IVM-containing solutions were applied via a rapid perfusion system (RSC-200, BIOLOGIC, Claix, France) consisting of an array of 5 glass tubes each approximately 400 μm in diameter. The application tube was routinely positioned at about 500 μm distance and about 50 μm above the recorded cell. A complete change of the solution around the cell took between 5–20 ms, depending on the speed of the solution expelled.

### Mathematical models

The one-layer Markov state model developed here (S6 Fig), is a revised version of a previously developed Markov model describing P2X4R orthosteric activation by ATP and allosteric modulation by IVM [20], whereas the two-layer model (Fig 3) is more complex in nature, taking into consideration all processes involved in ATP and IVM binding. Both models assume the presence of three ATP and three IVM binding sites and were tested against current recordings to compare their performance in capturing the physiological properties of P2X4R. The following symbols were used to describe the various states of the model: *C* for closed, *Q* for conducting (open_1_ and open_2_), *D* for desensitized and *Z* for internalized states, each representing the fraction of receptors in a given state. The transition rates between the various states are in Tables A and C in S1 Text. Detailed balance was not explicitly incorporated into these models because it was assumed that P2X4R never reach absolute equilibrium during ATP and IVM stimulation. Stimulation with ATP during the pulse protocol (repetitive stimulation with 1 μM ATP for 2 s twice per minute in the absence and presence of various concentrations of IVM applied to the bath medium after two ATP pulses) and the prolonged protocol (stimulation with 100 μM ATP for extended periods, longer than 1 min, in the absence and presence of 3 μM IVM) were modeled as a square wave and a rectangular function for the duration of ATP application, respectively; stimulation with IVM was modeled as a rectangular function for the duration of IVM application. The ramp protocol was modeled as a sawtooth-like sequence of upstrokes with slope 320 mV/s (rising from −80 mV +80 mV over 500 ms). Detailed descriptions of the two Markov models along with their differential equations are provided in Appendices A and B in S1 Text.

### Data characterization

Concentration-response data points were fit to a hill function

$$y=\frac{I_{\text{max}}}{1+{\left({\text{EC}}_{\text{50}}/x\right)}^{n}},$$

where *y* is the amplitude of the current evoked at a particular ATP concentration *x*, *I*_max_ is the maximum current observed at 100 μM ATP, EC_50_ is the ATP concentration producing 50% of the maximum current, and *n* is the Hill coefficient. Deactivation kinetics of the current decay after agonist washout were fitted to a single exponential

$$y=A_{1}\text{exp}\left(-t/\tau_{1}\right)+C$$

or to a sum of two exponentials

$$y=A_{1}\text{exp}\left(-t/\tau_{1}\right)+A_{2}\text{exp}\left(-t/\tau_{2}\right)+C,$$

where *A*_1_ and *A*_2_ are the amplitudes of decay for the first and second exponentials, *τ*_1_ and *τ*_2_ are their decay time constants, and *C* is the baseline current. In the case where the sum of exponentials fits the data better than a single exponential, we report the weighted time constant

$$\tau_{off}=\frac{A_{1}\tau_{1}+A_{2}\tau_{2}}{A_{1}+A_{2}}.$$

In either case, we labeled the derived time constant of deactivation as *τ*_*off*_. Statistical significance (**p<0.01 and *p<0.05) was assessed using the Wilcoxon signed rank test. MATLAB (MathWorks, Natick, MA) was used to solve the differential equations of the models numerically, fit the models to the data and apply statistical tests.

In order to quantify the rate of desensitization from current traces of the pulse protocol (see Figs 1 and 4) which do not show complete desensitization, we note that, at low concentrations of ATP (1 μM), desensitization is a mono-exponential process and thus employ a mono-exponential model

$$I(t)=Ae^{-\frac{t}{\tau }},$$

where *A* is the magnitude of the current at the onset of desensitization and *τ* is the time constant of desensitization. By first normalizing the current and then evaluating its derivative, with respect to time, at the onset of desensitization (t = 0), we obtain

$$\frac{\dot{I}(0)}{A}=-\frac{1}{\tau }.$$

Thus the first derivative at time *t* = 0 of each desensitizing current, $\dot{I}(0)$, normalized by its maximum current (as has been plotted in Fig 1B), yields information about the time constant of desensitization (i.e., a small normalized initial desensitization rate corresponds to a large time constant of desensitization). Due to the simultaneous activation and desensitization of multiple receptors, $\dot{I}(0)$ was estimated using a linear fit for the small window of time (1–1.5 s) after cells achieved their maximal current and before agonist was removed. This window is relatively small compared to the 6 s time constant of desensitization for P2X4R, and therefore this approximation method provides a relevant estimate of $\dot{I}(0)/A$, which serves as a proxy for desensitization time constant *τ*.

### Parameter estimation (Adaptive Parallel Tempering & t-Walk)

Parameter estimation was performed using MCMC techniques. Model simulations were generated using ode solvers in MATLAB and then fit to experimental recordings. Generally, MCMC produces Markov chains Λ = {**x**_1,_ **x**_2_, ⋯, **x**_*M*_} of model parameters $x_{m}=\left(p_{1}^{m},p_{2}^{m},\cdots ,p_{N}^{m}\right)$, where *N* is the number of parameters (*p*_*i*_) and *m* = 1, 2, ⋯, *M* is the *m*^th^ iterate of the Markov chain. The iterates represent samples from the posterior distribution *π*(**x**) determined using Bayes’ theorem as follows

π(x)∝L(x)P(x),

where L(**x**) = *P*(*data* | **x**), the likelihood function, is the probability of observing the data given the parameter values of **x**, and *P*(**x**) is the prior distribution of **x**, which reflects any prior knowledge about the parameter values independent of observed data. Proportionality, indicated by ∝, is sufficient—therefore there is no need to normalize the posterior.

In order to increase mixing of modes in parameter space, we used the parallel tempering algorithm which produces Markov chains in the product space

$$X_{m}\;=\;\{x_{m}^{\left(1\right)},\;x_{m}^{\left(2\right)},\cdots ,x_{m}^{\left(L\right)}\},$$

where each chain $x_{m}^{\left(l\right)}$, *l* = 1, 2, ⋯, *L*, was sampled from a tempered distribution $\pi^{\beta^{\left(l\right)}}$ and *β*^(*l*)^ is the inverse temperature of each chain. Parameter sets were stochastically swapped between chains according to the swap kernel of Miasojedow et al., and their strategy of adaptively updating the inverse temperature of each chain [33] was adopted. Because Metropolis-Hasting move-kernels can be difficult to tune for continuous-time Markov models of ion channels [34], we used the adaptive move kernel of the t-walk sampler [35] instead. Since the t-walk samples from the product distribution *π*(**x**)*π*(**x*′***), the composite MCMC method samples from the product distribution

$$\pi_{\beta }(X)\pi_{\beta }(X\prime )=\frac{\pi^{\beta^{\left(1\right)}}(x^{\left(1\right)})\pi^{\beta^{\left(1\right)}}(x\prime^{\left(1\right)})}{\int \pi^{\beta^{\left(1\right)}}(x)\pi^{\beta^{\left(1\right)}}(x\prime )dxdx\prime }\times \ldots \times \frac{\pi^{\beta^{\left(L\right)}}(x^{\left(L\right)})\pi^{\beta^{\left(L\right)}}(x\prime^{\left(L\right)})}{\int \pi^{\beta^{\left(L\right)}}(x)\pi^{\beta^{\left(L\right)}}(x\prime )dxdx\prime }.$$

Given that we have a set of discretely sampled whole-cell current recordings, we initially adopted the likelihood function from Gregory [36], defined by

$$\text{L}(x)=P(I_{\mu },\sigma |x)=\text{exp}\left\{-\overset{K}{\underset{i=1}{\sum }}\frac{{\left(I_{\mu }{}_{,i}-I_{x}{}_{,i}\right)}^{2}}{2\sigma_{i}^{2}}\right\},$$

where the index *i* refers to the *i*^th^ discretely sampled data point, *K* is the number of data points in the experimental recording, *I*_*μ*,*i*_ and *σ*_*i*_ are the *i*^th^ samples, respectively, of the mean current and current standard deviation estimated from the data set, and *I*_**x**,*i*_ is the *i*^th^ sample of the current produced by the model given the set of parameters **x**. To circumvent (i) sampling inefficiency from the posterior distribution, which is exacerbated by the use of high data sampling rates, and (ii) very poor fitting of rapid transient behaviour, due to the value of the likelihood being dominated by slower portions of the signal with more data points, we opted to fit (using the least-squares method) both experimental and simulation data to appropriately chosen functions and to compare the fit parameters of the experimental and simulated data. For example, we have used exponential functions to measure deactivation kinetics (as described above). This results in the likelihood function

$$\text{L}(x)=P(\tau_{off,\mu },\sigma_{\tau },A_{off,\mu },\sigma_{A}|x)=\text{exp}\left\{-\frac{{\left(\tau_{off,\mu }-\tau_{off,x}\right)}^{2}}{2\sigma_{\tau }^{2}}-\frac{{\left(A_{off,\mu }-A_{off,x}\right)}^{2}}{2\sigma_{A}^{2}}\right\},$$ (7)

where *τ*_*off*_,_*μ*_ and *σ*_*τ*_ are the mean deactivation time constant and its variance, respectively, *A*_*off*,*μ*_ and *σ*_*τ*_ are the mean deactivation amplitude and its variance, respectively, and *τ*_*off*_,_**x**_ and *A*_*off*_,_**x**_ are the deactivation time constant and amplitude produced by the model corresponding to the parameter values **x**.Using this description-based approach (rather than the distance from data), we were able to simultaneously fit numerous aspects of P2X4R activation kinetics and their allosteric modulation. This was done by comparing experimental data and model predictions of (*i*) time dependence of the activation time and normalized rate of desensitzation in the absence and presence of 1 μm IVM (Fig 1A and 1B) (*ii*) maximal current, deactivation time constant, and desensitization at 1 μM ATP with increasing IVM concentrations (Fig 4E and 4F) (iii). Insensitivity to ATP removal at 10 μM IVM (Fig 4H) (*iv*) decay of current amplitude deactivation time constant following IVM washout (Fig 4A–4D) (*v*) activation, desensitization, and recovery after washout of 100 μM ATP in the absence and presence of IVM (Fig 5C and 5D) (*vi*) EC_50_ and Hill coefficient (n) of the ATP concentration-response curves for peak current (Fig 5A).

The degree of cooperativity in ATP binding was determined from the Markov chain **x**_*m*_ of 5000 samples associated with each ATP binding and unbinding rate *k*_*i*_, *i* = 1, 2, ⋯, 24, that was generated from data fitting, followed by calculating the chains of ATP binding affinities

$$\frac{3k_{6n+2}}{k_{6}{}_{n+1}},\frac{k_{6n+4}}{k_{6}{}_{n+5}},\frac{k_{6n+6}}{3k_{6}{}_{n+5}},\;n=1,2,3$$

along each of the non-desensitized rows of the one-layer model (including the four lower rows) and the two-layer model (including the four rows in the upper layer). The posterior distributions associated with these affinities were used to compare the values of the most frequently sampled points along each row (*n* = 1, 2, 3). In the presence of a specific cooperativity between ATP bindings, correlations between the different binding affinities were detected and reported.

## Supporting information

S1 Text Supporting information. Model equations, parameter tables, supplemental experimental recordings and calculations.
(DOCX)

S1 Fig Shift in reversal potential associated with rat P2X4R expressed in HEK293 cells in the presence of IVM. Cells were bathed in extracellular solution containing: 155 mM NMDG^+^, 3 mM KCl, 2 mM CaCl_2_, 1 mM MgCl_2_, 10 mM HEPES and 10 mM D-glucose, adjusted to pH 7.3 with HCl. Patch electrodes were filled with solution containing 145 mM NaCl, 10 mM EGTA, and 10 mM HEPES. I-V curves showing how IVM shifts reversal potential during voltage ramp experiments. Substituting Na^+^ by NMDG^+^ in the medium does not change reversal potential in the absence of IVM (left) but shifts it from −33 mV to −13 mV in cells pretreated with 3 μM IVM for 30–60 s (right). In both cases, cells are stimulated with 100 μM ATP for 10 s and the voltage is ramped from −80 to +80 mV twice per second. A decrease in the total conductance of P2X4R (manifested as a decrease in the slope of the I-V curves) is observed under both conditions. Traces shown are representative of 30 similar experiments. Figure adapted with permission from [20].
(TIF)

S2 Fig Changes in conductance of IVM primed rat P2X4R. Currents induced by 100 μM ATP were recorded at a −60-mV holding potential during 60-s agonist application in the absence (left) and presence of IVM (right). In contrast to the recordings in S1 Fig (which were performed in the presence of Ca^2+^), the recordings presented here were performed in the absence of extracellular Ca^2+^, making the reversal potential for ATP-induced current more negative (about −70 mV [27]) than that in S1 Fig.
(TIF)

S3 Fig ATP-dependent concentration-response curves of peak current amplitude in control (blue circles) and 3 μM IVM primed cells (maroon and green circles). The cells were pretreated with IVM for 10 s (maroon circles) or for 30 s (green circles). Red arrow indicates the magnitude of the shift in the EC_50_. Data shown are mean±SEM values from n = 4–20 cells per dose. Figure adapted with permission from [20].
(TIF)

S4 Fig Rescue from desensitization and inactivation upon IVM application. A 50-s application with 100 μM ATP is used to desensitize nearly all P2X4R, followed by two 10-s ATP applications separated by 40-s washout periods demonstrating minimal recovery during washout. The subsequent addition of 3 μM IVM extracellularly in combination with 10-s ATP applications reveal an increase in receptor activation that persists even after IVM is removed 145 s later.
(TIF)

S5 Fig Enhanced desensitization (A) and inactivation (B), associated with the gating schemes shown in Fig 2, modify concentration-response curves of peak current amplitude in distinct ways. Hill functions were fit to ATP-dependent concentration-response curves of peak current amplitude generated using the gating schemes in Fig 2A and 2B, respectively. Enhanced desensitization and inactivation were realized by altering the magnitude of *k*_*d*_ in A and *H*_1_ in B, respectively, by a factor indicated in the legends. The fitted parameters of each Hill function (including I_max_, EC_50_ and *n*) for both cases are also presented in the legends. Enhanced desensitization increases EC_50_ and *n*, whereas enhanced inactivation decreases I_max_. Note that the range of agonist concentration along the *x*-axis spans 6 orders of magnitude but all responses in B reach saturation within 2 orders of magnitude, as observed experimentally (compare to S3 Fig), unlike in panel A.
(TIF)

S6 Fig A diagram of the one-layer Markov state model. The model describes the sequential binding and unbinding of ATP (denoted by *A*) along each row (desensitized, naïve, primed-1, primed-2 and primed-3), and IVM (denoted by *J*) along each column (below the naïve row). There are three ATP and three IVM binding sites; for details see Fig 3B. The states *C*_*i*_, *D*_*j*_ and *Q*_*i*_, *i* = 1, 2, ⋯, 8 and *j* = 1, 2, 3, 4, correspond to the fraction of receptors in the closed, desensitized and conducting states, respectively, and the states *C*_0_ and *Z* correspond to the fraction of receptors in the deeply inactivated and internalized states, respectively. The states *Q*_1_, *Q*_2_, *Q*_3_ and *Q*_4_ are open with maximum conductance *g*_1_ and reversal potential *E*_1_, whereas the primed states *Q*_5_, *Q*_6_, *Q*_7_ and *Q*_8_ are open with maximum conductance *g*_2_ > *g*_1_ and reversal potential *E*_2_ > *E*_1_. Transitions between states along rows represent binding (rightward) and unbinding (leftward) of one ATP molecule, and along columns (below the naïve row) represent the binding (downward) and unbinding (upward) of one IVM molecule. Before stimulation with ATP and IVM, receptors reside in either *C*_0_ or *C*_1_. Primed receptors must transition through the naïve states to desensitize, which is unlikely. The two-layer model (Fig 3A) removes this constraint. Parameter values of the one-layer model are listed in Table A.
(TIF)

S7 Fig Outcomes of the one-layer model as determined by the MCMC fitting to the pulse and prolonged protocols. (A-D) Time series of the current in the pulse protocol performed with (A) 0, (B) 1, (C) 3, and (D) 10 μM IVM. Dashed blue lines, experimental recordings; solid red lines, model simulations. (E, F) IVM-dependent concentration-response curves of (E) peak current amplitude and (F) rate of receptor deactivation. Model deactivation kinetics *τ*_*off*_ are measured by the weighted time constant. (G, H) Progression of activation, desensitization, and deactivation of currents produced by the model when normalized by maximum amplitudes during the pulse protocol in the presence of 1 μM (G) and 10 μM (H) IVM. All experimental data are derived from [20].
(TIF)

S8 Fig Outcomes of the one-layer model as determined by the MCMC fitting to the pulse and prolonged protocols. (A) ATP-dependent dose-response curves in the absence (black) and presence (red) of 3 μM IVM applied 30 s before ATP stimulation. Calculated EC_50_ and Hill coefficients in the absence of IVM were 1.7306 μM and 1.4244, respectively, and in the presence of IVM were 0.2386 μM and 1.2465, respectively. Dotted lines are experimental data. (B) The first and last I-V curves showing the decrease in their slopes and a positive shift in reversal potential upon stimulation with 100 μM ATP for 10 s in the presence of 3 μM IVM applied 20 s before ATP stimulation. The bath medium had Na^+^ replaced by NMDG^+^ modeled by setting *E*_1_ = −46.1 mV and *E*_2_ = −21.9 mV. The voltage is ramped from −80 mV to +80 mV twice per second from a holding potential of −60 mV. (C), Two prolonged applications of 100 μM ATP produced by the model in the absence of IVM separated by a 3 min washout period. (D) Prolonged application of 100 μM ATP produced by the model in the presence of 3 μM IVM. All experimental data are derived from [20].
(TIF)

S9 Fig Gating scheme of Fig 2A captures the short timescale behaviour during the pulse protocol. (A) Single gating scheme fitting (orange line) to the first pulse (before IVM application) of a 1 μM IVM pulse protocol recording (blue line) derived from experiments shown in S7B Fig. (B) The remainder of the current time series produced by the gating scheme used to fit the pulse in A. (C) Single gating scheme fitting (orange line) to the last pulse (before IVM washout) of a 10 μM IVM pulse protocol recording (blue line) derived from experiments shown in S7D Fig. (D) The remainder of the current time series produced by the gating scheme used to fit the pulse in (C). All experimental data are derived from [20].
(TIF)

S10 Fig Multiple experimental realizations of a prolonged application of 100 μM ATP in cells primed with 3 μM IVM. Experimental conditions are identical to those used to produce the data in Fig 5D.
(TIF)

S11 Fig Outcomes of the two-layer model, lacking unitary conductance increase (*g*_1_ = *g*_2_), determined by the MCMC fitting to the pulse and prolonged protocols. (A-D) Simulated time series of current for the pulse protocol performed with (A) 0, (B) 1, (C) 3, and (D) 10 μM IVM. Dashed blue lines, experimental recordings; solid red lines, simulations. (E, F) IVM-dependent concentration-response curves of (E) peak current amplitude and (F) rate of receptor deactivation. Model deactivation kinetics ***τ***_*off*_ measured by the weighted time constant. (G, H) Progression of activation, desensitization, and deactivation of currents produced by the model and normalized by maximum amplitudes during the pulse protocol in the presence of 1 μM (G) and 10 μM (H) IVM. Insets show the magnified desensitization phases of the response. All experimental data are derived from [20].
(TIF)

S12 Fig Outcomes of the two-layer, model lacking unitary conductance increase (*g*_1_ = *g*_2_), determined by the MCMC fitting to the pulse and prolonged protocols. (A) ATP-dependent concentration-response curves in the absence (black) and presence (red) of 3 μM IVM applied 30 s before ATP stimulation. Calculated EC_50_ and Hill coefficients in the absence of IVM were 1.6549 μM and 1.5777, respectively, and in the presence of IVM were 0.65606 and 0.88552 μM, respectively. Dotted lines are experimental data. (B) The first and last I-V curves showing the decrease in their slopes and a positive shift in reversal potential upon stimulation with 100 μM ATP for 10 s in the presence of 3 μM IVM applied 20 s before ATP stimulation. The bath medium had Na^+^ replaced by NMDG^+^ modeled by setting *E*_1_ = −107.8 mV and *E*_2_ = −12.3 mV. Voltage was ramped from −80 mV to +80 mV twice per second from a holding potential of −60 mV. (C) Two prolonged applications of 100 μM ATP separated by a 4 min washout period in the absence of IVM. (D) Prolonged application of 100 μM ATP produced by the model in the presence of 3 μM IVM. All experimental data were derived from [20].
(TIF)

## References

[1] Barrera N.P., et al, Atomic force microscopy imaging demonstrates that P2X2 receptors are trimers but that P2X6 receptor subunits do not oligomerize. Journal of Biological Chemistry, 2005 280(11): p. 10759–10765. doi: 10.1074/jbc.M412265200 15657042 10.1074/jbc.M412265200

[2] Hattori M. and Gouaux E., Molecular mechanism of ATP binding and ion channel activation in P2X receptors. Nature, 2012 485(7397): p. 207–212. doi: 10.1038/nature11010 22535247 10.1038/nature11010 PMC3391165

[3] Kawate T., et al, Crystal structure of the ATP-gated P2X4 ion channel in the closed state. Nature, 2009 460(7255): p. 592–598. doi: 10.1038/nature08198 19641588 10.1038/nature08198 PMC2720809

[4] Mio K., et al, Visualization of the trimeric P2X 2 receptor with a crown-capped extracellular domain. Biochemical and biophysical research communications, 2005 337(3): p. 998–1005. doi: 10.1016/j.bbrc.2005.09.141 16219297 10.1016/j.bbrc.2005.09.141

[5] Nicke A., et al, P2X1 and P2X3 receptors form stable trimers: a novel structural motif of ligand-gated ion channels. The EMBO journal, 1998 17(11): p. 3016–3028. doi: 10.1093/emboj/17.11.3016 9606184 10.1093/emboj/17.11.3016 PMC1170641

[6] Kaczmarek-Hájek K., et al, Molecular and functional properties of P2X receptors—recent progress and persisting challenges. Purinergic Signalling, 2012 8(3): p. 375–417. doi: 10.1007/s11302-012-9314-7 22547202 10.1007/s11302-012-9314-7 PMC3360091

[7] Marquez-Klaka B., et al, Identification of an intersubunit cross-link between substituted cysteine residues located in the putative ATP binding site of the P2X1 receptor. The Journal of neuroscience, 2007 27(6): p. 1456–1466. doi: 10.1523/JNEUROSCI.3105-06.2007 17287520 10.1523/JNEUROSCI.3105-06.2007 PMC6673578

[8] Migita K., et al, Polar Residues of the Second Transmembrane Domain Influence Cation Permeability of the ATP-gated P2X2 Receptor. Journal of Biological Chemistry, 2001 276: p. 30934–30941. doi: 10.1074/jbc.M103366200 11402044 10.1074/jbc.M103366200

[9] Coddou C., et al, Activation and regulation of purinergic P2X receptor channels. Pharmacological reviews, 2011 63(3): p. 641–683. doi: 10.1124/pr.110.003129 21737531 10.1124/pr.110.003129 PMC3141880

[10] Roberts J.A. and Evans R.J., ATP binding at human P2X1 receptors Contribution of aromatic and basic amino acids revealed using mutagenesis and partial agonists. Journal of Biological Chemistry, 2004 279(10): p. 9043–9055. doi: 10.1074/jbc.M308964200 14699168 10.1074/jbc.M308964200

[11] Egan T.M., Samways D.S.K., and Li Z., Biophysics of P2X receptors. Pflügers Archiv, 2006 452(5): p. 501–512. doi: 10.1007/s00424-006-0078-1 16708237 10.1007/s00424-006-0078-1

[12] Virginio C., et al, Kinetics of cell lysis, dye uptake and permeability changes in cells expressing the rat P2X7 receptor. The Journal of physiology, 1999 519(2): p. 335–346. 10457053 10.1111/j.1469-7793.1999.0335m.x PMC2269518

[13] Li M., et al, Physical basis of apparent pore dilation of ATP-activated P2X receptor channels. Nature neuroscience, 2015. 10.1038/nn.4120 PMC5113834 26389841

[14] Wei L., et al, ATP-induced P2X Receptor-Dependent Large Pore Formation: How Much Do We Know? Frontiers in Pharmacology, 2016 7(5). 10.3389/fphar.2016.00005 PMC4732382 26858647

[15] Harkat M., et al, On the permeation of large organic cations through the pore of ATP-gated P2X receptors. Proceedings of the National Academy of Sciences, 2017 114(19): p. E3786–E3795. 10.1073/pnas.1701379114 PMC5441707 28442564

[16] Khakh B.S., et al, Allosteric control of gating and kinetics at P2X(4) receptor channels. J Neurosci, 1999 19(17): p. 7289–99. 10460235 10.1523/JNEUROSCI.19-17-07289.1999 PMC6782529

[17] Priel A. and Silberberg S.D., Mechanism of ivermectin facilitation of human P2X4 receptor channels. J Gen Physiol, 2004 123(3): p. 281–93. doi: 10.1085/jgp.200308986 14769846 10.1085/jgp.200308986 PMC2217454

[18] Jelinkova I., et al, Identification of P2X(4) receptor-specific residues contributing to the ivermectin effects on channel deactivation. Biochem Biophys Res Commun, 2006 349(2): p. 619–25. doi: 10.1016/j.bbrc.2006.08.084 16949036 10.1016/j.bbrc.2006.08.084

[19] Khadra A., et al, Gating properties of the P2X2a and P2X2b receptor channels: experiments and mathematical modeling. The Journal of general physiology, 2012 139(5): p. 333–348. doi: 10.1085/jgp.201110716 22547664 10.1085/jgp.201110716 PMC3343373

[20] Zemkova H., et al, Allosteric regulation of the P2X4 receptor channel pore dilation. Pflügers Archiv-European Journal of Physiology, 2015 467(4): p. 713–726. doi: 10.1007/s00424-014-1546-7 24917516 10.1007/s00424-014-1546-7 PMC4526220

[21] Zemkova H., et al, Role of aromatic and charged ectodomain residues in the P2X4 receptor functions. Journal of Neurochemistry, 2007 102(4): p. 1139–1150. doi: 10.1111/j.1471-4159.2007.04616.x 17663752 10.1111/j.1471-4159.2007.04616.x

[22] Hibbs R.E. and Gouaux E., Principles of activation and permeation in an anion-selective Cys-loop receptor. Nature, 2011 474(7349): p. 54–60. doi: 10.1038/nature10139 21572436 10.1038/nature10139 PMC3160419

[23] Silberberg S.D., Li M., and Swartz K.J., Ivermectin interaction with transmembrane helices reveals widespread rearrangements during opening of P2X receptor channels. Neuron, 2007 54(2): p. 263–274. doi: 10.1016/j.neuron.2007.03.020 17442247 10.1016/j.neuron.2007.03.020

[24] Yan Z., et al, Experimental characterization and mathematical modeling of P2X7 receptor channel gating. The Journal of Neuroscience, 2010 30(42): p. 14213–14224. doi: 10.1523/JNEUROSCI.2390-10.2010 20962242 10.1523/JNEUROSCI.2390-10.2010 PMC2980950

[25] Yan Z., et al, Calcium-dependent block of P2X7 receptor channel function is allosteric. The Journal of general physiology, 2011 138(4): p. 437–452. doi: 10.1085/jgp.201110647 21911484 10.1085/jgp.201110647 PMC3182445

[26] Khadra A., et al, Dual gating mechanism and function of P2X7 receptor channels. Biophysical journal, 2013 104(12): p. 2612–2621. doi: 10.1016/j.bpj.2013.05.006 23790369 10.1016/j.bpj.2013.05.006 PMC3686336

[27] Yan Z., et al, The P2X(7) Receptor Channel Pore Dilates under Physiological Ion Conditions. The Journal of General Physiology, 2008 132(5): p. 563–573. doi: 10.1085/jgp.200810059 18852304 10.1085/jgp.200810059 PMC2571973

[28] Browne L.E. and North R.A., P2X Receptor Intermediate Activation States Have Altered Nucleotide Selectivity. The Journal of Neuroscience, 2013 33(37): p. 14801–14808. doi: 10.1523/JNEUROSCI.2022-13.2013 24027280 10.1523/JNEUROSCI.2022-13.2013 PMC3771025

[29] Mackay L., et al, Deciphering the Kinetic and Gating Properties of Purinergic P2X7 Receptor Channels. Athens Journal of Natural & Formal Sciences, 2014 1(1).

[30] Vandenberg C. and Bezanilla F., A sodium channel gating model based on single channel, macroscopic ionic, and gating currents in the squid giant axon. Biophysical journal, 1991 60(6): p. 1511 doi: 10.1016/S0006-3495(91)82186-5 1663796 10.1016/S0006-3495(91)82186-5 PMC1260209

[31] Toulmé E., et al, Functional properties of internalization-deficient P2X4 receptors reveal a novel mechanism of ligand-gated channel facilitation by ivermectin. Molecular pharmacology, 2006 69(2): p. 576–587. doi: 10.1124/mol.105.018812 16282518 10.1124/mol.105.018812

[32] Habermacher C., et al, Photo-switchable tweezers illuminate pore-opening motions of an ATP-gated P2X ion channel. eLife, 2016 5: p. e11050 doi: 10.7554/eLife.11050 26808983 10.7554/eLife.11050 PMC4739762

[33] Miasojedow B., Moulines E., and Vihola M., An adaptive parallel tempering algorithm. Journal of Computational and Graphical Statistics, 2013 22(3): p. 649–664.

[34] Siekmann I., Sneyd J., and Crampin E.J., MCMC can detect nonidentifiable models. Biophysical journal, 2012 103(11): p. 2275–2286. doi: 10.1016/j.bpj.2012.10.024 23283226 10.1016/j.bpj.2012.10.024 PMC3514526

[35] Christen J.A. and Fox C., A general purpose sampling algorithm for continuous distributions (the t-walk). Bayesian Analysis, 2010 5(2): p. 263–281.

[36] Gregory P., *Bayesian Logical Data Analysis for the Physical Sciences*: *A Comparative Approach with Mathematica*^®^*Support*. 2005: Cambridge University Press.

