# Supplementary material for: Deciphering the regulation of P2X4 receptor channel gating by ivermectin using Markov models
Source: PLoS Comput Biol. 2017 Jul 14;13(7):e1005643. doi: 10.1371/journal.pcbi.1005643 (PMC5533465; doi:10.1371/journal.pcbi.1005643)
Supplement: S1 Text — Model equations, parameter tables, supplemental experimental recordings and calculations. (DOCX) [file pcbi.1005643.s001.docx]

**Supporting Information**

**Table A.** Parameter values of the one-layer model determined using MCMC techniques. The reversal potentials $E_{1}$ and $E_{2}$ are zero under symmetric ionic conditions, and take the values listed below in NMDG^+^ containing media in the presence of IVM.

| Symbol | Value | Unit | Transitions | Symbol | Value | Unit | Transitions |
| --- | --- | --- | --- | --- | --- | --- | --- |
| $k_{1}$ | 3.806 | s^-1^ | $C_{2},D_{2}\to C_{1},D_{1}$ | $L_{11}$ | 1.598$\times10^{-3}$ | s^-1^ | $C_{3}\to C_{1}$ |
| $k_{2}$ | 8.946$\times10^{5}$ | (s∙M)^-1^ | $C_{1},D_{11}\to C_{2},D_{2}$ | $L_{12}$ | 1.055$\times10^{-2}$ | s^-1^ | $C_{4}\to C_{2}$ |
| $k_{3}$ | 2.522 | s^-1^ | $Q_{1},D_{3}\to C_{2},D_{2}$ | $L_{13}$ | 7.496$\times10^{-1}$ | s^-1^ | $Q_{3}\to Q_{1}$ |
| $k_{4}$ | 3.528$\times10^{6}$ | (s∙M)^-1^ | $C_{2},D_{2}\to Q_{1},D_{3}$ | $L_{14}$ | 7.139$\times10^{-1}$ | s^-1^ | $Q_{4}\to Q_{2}$ |
| $k_{5}$ | 14.76 | s^-1^ | $Q_{2},D_{4}\to Q_{1},D_{3}$ | $L_{21}$ | 4.617$\times10^{-1}$ | s^-1^ | $C_{1}\to C_{3}$ |
| $k_{6}$ | 2.765$\times10^{5}$ | (s∙M)^-1^ | $Q_{1},D_{3}\to Q_{2},D_{4}$ | $L_{22}$ | 2.937$\times10^{-1}$ | s^-1^ | $C_{2}\to C_{4}$ |
| $k_{7}$ | 2.213$\times10^{-3}$ | s^-1^ | $C_{4}\to C_{3}$ | $L_{23}$ | 2.259$\times10^{-1}$ | s^-1^ | $Q_{1}\to Q_{3}$ |
| $k_{8}$ | 1.379$\times10^{6}$ | (s∙M)^-1^ | $C_{3}\to C_{4}$ | $L_{24}$ | 2.536$\times10^{-1}$ | s^-1^ | $Q_{2}\to Q_{4}$ |
| $k_{9}$ | 2.576 | s^-1^ | $Q_{3}\to C_{4}$ | $L_{31}$ | 4.561$\times10^{-2}$ | s^-1^ | $C_{5}\to C_{3}$ |
| $k_{10}$ | 6.630$\times10^{6}$ | (s∙M)^-1^ | $C_{4}\to Q_{3}$ | $L_{32}$ | 2.376$\times10^{-3}$ | s^-1^ | $C_{6}\to C_{4}$ |
| $k_{11}$ | 1.849 | s^-1^ | $Q_{4}\to Q_{3}$ | $L_{33}$ | 4.437$\times10^{-3}$ | s^-1^ | $Q_{5}\to Q_{3}$ |
| $k_{12}$ | 1.404$\times10^{7}$ | (s∙M)^-1^ | $Q_{3}\to Q_{4}$ | $L_{34}$ | 8.184$\times10^{-3}$ | s^-1^ | $Q_{6}\to Q_{4}$ |
| $k_{13}$ | 2.179$\times10^{-3}$ | s^-1^ | $C_{6}\to C_{5}$ | $L_{41}$ | 1.725$\times10^{-2}$ | s^-1^ | $C_{3}\to C_{5}$ |
| $k_{14}$ | 1.179$\times10^{6}$ | (s∙M)^-1^ | $C_{5}\to C_{6}$ | $L_{42}$ | 4.472$\times10^{-1}$ | s^-1^ | $C_{4}\to C_{6}$ |
| $k_{15}$ | 4.114$\times10^{-1}$ | s^-1^ | $Q_{5}\to C_{6}$ | $L_{43}$ | 8.250$\times10^{-3}$ | s^-1^ | $Q_{3}\to Q_{5}$ |
| $k_{16}$ | 8.805$\times10^{6}$ | (s∙M)^-1^ | $C_{6}\to Q_{5}$ | $L_{44}$ | 2.033$\times10^{-1}$ | s^-1^ | $Q_{4}\to Q_{6}$ |
| $k_{17}$ | 2.264$\times10^{-1}$ | s^-1^ | $Q_{6}\to Q_{5}$ | $L_{51}$ | 5.122$\times10^{-3}$ | s^-1^ | $C_{7}\to C_{5}$ |
| $k_{18}$ | 8.690$\times10^{6}$ | (s∙M)^-1^ | $Q_{5}\to Q_{6}$ | $L_{52}$ | 2.742$\times10^{-2}$ | s^-1^ | $C_{8}\to C_{6}$ |
| $k_{19}$ | 7.997$\times10^{-3}$ | s^-1^ | $C_{8}\to C_{7}$ | $L_{53}$ | 2.490$\times10^{-2}$ | s^-1^ | $Q_{7}\to Q_{5}$ |
| $k_{20}$ | 1.257$\times10^{7}$ | (s∙M)^-1^ | $C_{7}\to C_{8}$ | $L_{54}$ | 9.690$\times10^{-2}$ | s^-1^ | $Q_{8}\to Q_{6}$ |
| $k_{21}$ | 3.018$\times10^{-4}$ | s^-1^ | $Q_{7}\to C_{8}$ | $L_{61}$ | 4.074$\times10^{-1}$ | s^-1^ | $C_{5}\to C_{7}$ |
| $k_{22}$ | 2.390$\times10^{6}$ | (s∙M)^-1^ | $C_{8}\to Q_{7}$ | $L_{62}$ | 4.688$\times10^{-1}$ | s^-1^ | $C_{6}\to C_{8}$ |
| $k_{23}$ | 3.018$\times10^{-4}$ | s^-1^ | $Q_{8}\to Q_{7}$ | $L_{63}$ | 2.724$\times10^{-2}$ | s^-1^ | $Q_{5}\to Q_{7}$ |
| $k_{24}$ | 5.820$\times10^{6}$ | (s∙M)^-1^ | $Q_{7}\to Q_{8}$ | $L_{64}$ | 3.188$\times10^{-2}$ | s^-1^ | $Q_{6}\to Q_{8}$ |
| $k_{d}$ | 9.021$\times10^{-3}$ | s^-1^ | $\begin{matrix} C_{2},Q_{1},Q_{2}\to\\ D_{2},D_{3},D_{4} \end{matrix}$ | $k_{r}$ | 2.495$\times10^{-7}$ | s^-1^ | $\begin{matrix} D_{1}, D_{2}, D_{3}, D_{4}\to\\ C_{1}, C_{2}, Q_{1}, Q_{2} \end{matrix}$ |
| $H_{1}$ | 9.511$\times10^{-4}$ | s^-1^ | $C_{0}\to C_{1}$ | $H_{2}$ | 1.196$\times10^{-3}$ | s^-1^ | $C_{1}\to C_{0}$ |
| $H_{3}$ | 6.811$\times10^{-3}$ | s^-1^ | $D_{4}\to Z$ | $H_{4}$ | 5.320$\times10^{-4}$ | s^-1^ | $Z\to C_{0}$ |
| $L$ | 8.981$\times10^{-1}$ | s^-1^ | $C_{0}\to C_{1}$ | $\kappa_{1}$ | 1.300$\times10^{-6}$ | M | $C_{0},C_{1},C_{2},Q_{1},Q_{2}\to$ $C_{3},C_{3},C_{4},Q_{3},Q_{4}$ |
| $\kappa_{2}$ | 1.775$\times10^{-6}$ | M | $C_{3},C_{4},Q_{3},Q_{4}\to$ $C_{5},C_{6},Q_{5},Q_{6}$ | $\kappa_{3}$ | 3.686$\times10^{-6}$ | M | $C_{5},C_{6},Q_{5},Q_{6}\to$ $C_{7},C_{8},Q_{7},Q_{8}$ |
| $g_{1}$ | 6.8281$\times10^{-8}$ | S | - | $g_{2}$ | 6.8820$\times10^{-8}$ | S | - |
| $E_{1}$ | -46.1 | mV | - | $E_{2}$ | -21.9 | mV | - |

**Table B.** A list of modifications made to the transition rates of ATP binding and unbinding.

| Original Parameter | Modified Expression | Original Parameter | Modified Expression | Original Parameter | Modified Expression |
| --- | --- | --- | --- | --- | --- |
| $k_{7}$ | $v_{1}k_{1}$ | $k_{13}$ | $v_{2}k_{1}$ | $k_{19}$ | $v_{3}k_{1}$ |
| $k_{8}$ | $r_{1}k_{2}$ | $k_{14}$ | $r_{2}k_{2}$ | $k_{20}$ | $r_{3}k_{2}$ |
| $k_{9}$ | $v_{1}k_{3}$ | $k_{15}$ | $v_{2}k_{3}$ | $k_{21}$ | $v_{3}k_{3}$ |
| $k_{10}$ | $r_{1}k_{4}$ | $k_{16}$ | $r_{2}k_{4}$ | $k_{22}$ | $r_{3}k_{4}$ |
| $k_{11}$ | $v_{1}k_{5}$ | $k_{17}$ | $v_{2}k_{5}$ | $k_{23}$ | $v_{3}k_{5}$ |
| $k_{12}$ | $r_{1}k_{6}$ | $k_{18}$ | $r_{2}k_{6}$ | $k_{24}$ | $r_{3}k_{6}$ |

**Table C:** Parameter values of the two-layer model determined using MCMC techniques. The reversal potentials $E_{1}$ and $E_{2}$ are zero under symmetric ionic conditions, and take the values listed below in NMDG^+^ containing media in the presence of IVM.

| Symbol | Value | Units | Transitions | Symbol | Value | Units | Transitions |
| --- | --- | --- | --- | --- | --- | --- | --- |
| $k_{1}$ | 12.28 | s^-1^ | $C_{2},D_{12}\to C_{1},D_{11}$ | $L_{11}$ | 2.530$\times10^{-2}$ | s^-1^ | $C_{3},D_{21}\to C_{1},D_{11}$ |
| $k_{2}$ | 2.282$\times10^{6}$ | (s∙M)^-1^ | $C_{1},D_{11}\to C_{2},D_{12}$ | $L_{12}$ | 3.938$\times10^{-2}$ | s^-1^ | $C_{4},D_{22}\to C_{2},D_{12}$ |
| $k_{3}$ | 6.134 | s^-1^ | $Q_{1},D_{13}\to C_{2},D_{12}$ | $L_{13}$ | 0.7299 | s^-1^ | $Q_{3},D_{23}\to Q_{1},D_{13}$ |
| $k_{4}$ | 1.752$\times10^{7}$ | (s∙M)^-1^ | $C_{2},D_{12}\to Q_{1},D_{13}$ | $L_{14}$ | 0.2680 | s^-1^ | $Q_{4},D_{24}\to Q_{2},D_{14}$ |
| $k_{5}$ | 2.658$\times10^{3}$ | s^-1^ | $Q_{2},D_{14}\to Q_{1},D_{13}$ | $L_{21}$ | 0.2508 | s^-1^ | $C_{1},D_{11}\to C_{3},D_{21}$ |
| $k_{6}$ | 1.319$\times10^{3}$ | (s∙M)^-1^ | $Q_{1},D_{13}\to Q_{2},D_{14}$ | $L_{22}$ | 0.1954 | s^-1^ | $C_{2},D_{12}\to C_{4},D_{22}$ |
| $k_{7}$ | 4.593$\times10^{-4}$ | s^-1^ | $C_{4},D_{22}\to C_{3},D_{21}$ | $L_{23}$ | 2.323$\times10^{-2}$ | s^-1^ | $Q_{1},D_{13}\to Q_{3},D_{23}$ |
| $k_{8}$ | 4.260$\times10^{6}$ | (s∙M)^-1^ | $C_{3},D_{21}\to C_{4},D_{22}$ | $L_{24}$ | 1.435 | s^-1^ | $Q_{2},D_{14}\to Q_{4},D_{24}$ |
| $k_{9}$ | 0.3530 | s^-1^ | $Q_{3},D_{23}\to C_{4},D_{22}$ | $L_{31}$ | 9.686$\times10^{-3}$ | s^-1^ | $C_{5},D_{32}\to C_{3},D_{21}$ |
| $k_{10}$ | 4.547$\times10^{7}$ | (s∙M)^-1^ | $C_{4},D_{22}\to Q_{3},D_{23}$ | $L_{32}$ | 7.485$\times10^{-3}$ | s^-1^ | $C_{6},D_{31}\to C_{4},D_{22}$ |
| $k_{11}$ | 0.7347 | s^-1^ | $Q_{4},D_{24}\to Q_{3},D_{23}$ | $L_{33}$ | 1.089$\times10^{-2}$ | s^-1^ | $Q_{5},D_{33}\to Q_{3},D_{23}$ |
| $k_{12}$ | 4.510$\times10^{6}$ | (s∙M)^-1^ | $Q_{3},D_{23}\to Q_{4},D_{24}$ | $L_{34}$ | 0.1069 | s^-1^ | $Q_{6},D_{34}\to Q_{4},D_{24}$ |
| $k_{13}$ | 6.448$\times10^{-2}$ | s^-1^ | $C_{6},D_{32}\to C_{5},D_{31}$ | $L_{41}$ | 3.300$\times10^{-2}$ | s^-1^ | $C_{3},D_{21}\to C_{5},D_{32}$ |
| $k_{14}$ | 3.929$\times10^{6}$ | (s∙M)^-1^ | $C_{5},D_{31}\to C_{6},D_{32}$ | $L_{42}$ | 0.5552 | s^-1^ | $C_{4},D_{22}\to C_{6},D_{31}$ |
| $k_{15}$ | 0.3094 | s^-1^ | $Q_{5},D_{33}\to C_{6},D_{32}$ | $L_{43}$ | 0.2177 | s^-1^ | $Q_{3},D_{23}\to Q_{5},D_{33}$ |
| $k_{16}$ | 1.921$\times10^{6}$ | (s∙M)^-1^ | $C_{6},D_{32}\to Q_{5},D_{33}$ | $L_{44}$ | 1.261 | s^-1^ | $Q_{4},D_{24}\to Q_{6},D_{34}$ |
| $k_{17}$ | 0.7425 | s^-1^ | $Q_{6},D_{34}\to Q_{5},D_{33}$ | $L_{51}$ | 2.226$\times10^{-4}$ | s^-1^ | $C_{7},D_{41}\to C_{5},D_{31}$ |
| $k_{18}$ | 1.505$\times10^{7}$ | (s∙M)^-1^ | $Q_{5},D_{33}\to Q_{6},D_{34}$ | $L_{52}$ | 0.1042 | s^-1^ | $C_{8},D_{42}\to C_{6},D_{32}$ |
| $k_{19}$ | 0.6376 | s^-1^ | $C_{8},D_{42}\to C_{7},D_{41}$ | $L_{53}$ | 0.1359 | s^-1^ | $Q_{7},D_{43}\to Q_{5},D_{33}$ |
| $k_{20}$ | 2.913$\times10^{7}$ | (s∙M)^-1^ | $C_{7},D_{41}\to C_{8},D_{42}$ | $L_{54}$ | 6.130$\times10^{-2}$ | s^-1^ | $Q_{8},D_{44}\to Q_{6},D_{34}$ |
| $k_{21}$ | 4.208$\times10^{-3}$ | s^-1^ | $Q_{7},D_{43}\to C_{8},D_{42}$ | $L_{61}$ | 1.631$\times10^{-2}$ | s^-1^ | $C_{5},D_{31}\to C_{7},D_{41}$ |
| $k_{22}$ | 1.919$\times10^{6}$ | (s∙M)^-1^ | $C_{8},D_{42}\to Q_{7},D_{43}$ | $L_{62}$ | 0.6876 | s^-1^ | $C_{6},D_{32}\to C_{8},D_{42}$ |
| $k_{23}$ | 3.514$\times10^{-3}$ | s^-1^ | $Q_{8},D_{44}\to Q_{7},D_{43}$ | $L_{63}$ | 8.935$\times10^{-2}$ | s^-1^ | $Q_{5},D_{33}\to Q_{7},D_{43}$ |
| $k_{24}$ | 3.724$\times10^{6}$ | (s∙M)^-1^ | $Q_{7},D_{43}\to Q_{8},D_{44}$ | $L_{64}$ | 1.075$\times10^{-3}$ | s^-1^ | $Q_{6},D_{34}\to Q_{8},D_{44}$ |
| $L$ | 9.001$\times10^{-2}$ | s^-1^ | $C_{0}\to C_{3}$ | $H_{1}$ | 1.623$\times10^{-3}$ | s^-1^ | $C_{0}\to C_{1}$ |
| $H_{2}$ | 1.900$\times10^{-3}$ | s^-1^ | $C_{1}\to C_{0}$ | $H_{3}$ | 0.1577 | s^-1^ | $D_{14}\to Z$ |
| $H_{4}$ | 2.305$\times10^{-2}$ | s^-1^ | $Z\to C_{0}$ | $H_{5}$ | 1.785$\times10^{-4}$ | s^-1^ | $D_{24},D_{34},D_{44}\to Z$ |
| $k_{d,1}$ | 0.1667 | s^-1^ | $C_{2},Q_{1},Q_{2}\to D_{12},D_{13},D_{14}$ | $k_{r,1}$ | 1.683$\times10^{-5}$ | s^-1^ | $D_{11},D_{12},D_{13},D_{14}\to$ $C_{1},C_{2},Q_{1},Q_{2}$ |
| $k_{d,2}$ | 0.9278 | s^-1^ | $C_{4},Q_{3},Q_{4}\to D_{22},D_{23},D_{24}$ | $k_{r,2}$ | 4.552 | s^-1^ | $D_{21},D_{22},D_{23},D_{24}\to$ $C_{3},C_{4},Q_{3},Q_{4}$ |
| $k_{d,3}$ | 0.5486 | s^-1^ | $C_{6},Q_{5},Q_{6}\to D_{32},D_{33},D_{34}$ | $k_{r,3}$ | 0.4896 | s^-1^ | $D_{31},D_{32},D_{33},D_{34}\to$ $C_{5},C_{6},Q_{5},Q_{6}$ |
| $k_{d,4}$ | 0.5299 | s^-1^ | $C_{8},Q_{7},Q_{8}\to D_{42},D_{43},D_{44}$ | $k_{r,4}$ | 0.5860 | s^-1^ | $\begin{matrix} D_{41},D_{42},D_{43},D_{44}\to\\ C_{7},C_{8},Q_{7},Q_{8} \end{matrix}$ |
| $\kappa_{1}$ | 9.904$\times10^{-7}$ | M | $\begin{matrix} C_{0},C_{1},C_{2},Q_{1},Q_{2},D_{11},D_{12},D_{13},D_{14}\to\\ C_{3},C_{3},C_{4},Q_{3},Q_{4},D_{21},D_{22},D_{23},D_{24} \end{matrix}$ | $g_{1}$ | 6.8236$\times10^{-8}$ | S | - |
| $\kappa_{2}$ | 1.744$\times10^{-6}$ | M | $\begin{matrix} C_{3},C_{4},Q_{3},Q_{4},D_{21},D_{22},D_{23},D_{24}\to\\ C_{5},C_{6},Q_{5},Q_{6},D_{32},D_{31},D_{33},D_{34} \end{matrix}$ | $g_{2}$ | 7.5896$\times10^{-8}$ | S | - |
| $\kappa_{3}$ | 6.755$\times10^{-6}$ | M | $\begin{matrix} C_{5},C_{6},Q_{5},Q_{6},D_{31},D_{32},D_{33},D_{34}\to\\ C_{7},C_{8},Q_{7},Q_{8},D_{41},D_{42},D_{43},D_{44} \end{matrix}$ | $E_{1}$ | -93.5 | mV | - |
| $E_{2}$ | -5.1 | mV | - |  |  |  |  |

**Appendix A**

**Description of the One-Layer Model**

We revised the model of Zemkova et. al. [1], by assuming that IVM acts on P2X4R independently of ATP binding, and allowing for IVM to induce sequential transitions from all states in the naïve row as opposed to only from the 3-ATP bound state. A deeply inactivated state $C_{0}$ rescued by IVM was also added (see below). Because there are three IVM binding sites of interaction on a single receptor, we also allow for IVM to transition to three additional rows (labled primed-1, primed-2 and primed-3 rows; see Fig S6). These rows possess modified kinetics and conductances to cature the various effects of IVM. In the most general case, the multiple IVM interaction sites are independent of one another. We therefore model such interactions as independent transitions from the naïve row and allow for further transitions between each IVM modified row (i.e., transitions between different columns).

Since the two effects associated with different IVM interaction sites are known to have distinct pharmacological profiles [1, 2], modeling the numerous IVM interactions as a sequential binding process not only simplifies the model and reduces the number of parameters, but also becomes justified. According to Fig S6, the transition rates of ATP unbinding/binding were denoted by $k_{1}, k_{2}, \cdots,k_{24}$ (where odd subscripts are associated with unbindings and even subscripts with binding), the transition rates of IVM binding by $L_{ij}f_{n}(J)$ ($i=2n, j=1, 2, 3, 4$ and $n=1, 2, 3$), where

$$f_{n}(J)=\frac{J^{4}}{\kappa_{n}^{4}+J^{4}}$$

($J=[\text{IVM}]$ and $\kappa_{n}$ are the EC_50_ of the 1^st^, 2^nd^ and 3^rd^ transitions induced by IVM binding, repspectively), the transition rates of IVM unbinding by $L_{ij}$ ($i=2n-1, j=1, 2, 3, 4$ and $n=1, 2, 3$), the transition rate for desensitization (recovery) by $k_{d}$ ($k_{r}$), and the transition rate of fully occupied naïve receptors for internalizaiton (recycling) by $H_{3}$ ($H_{4}$). To allow for the steady-state population to continuously shift towards the last IVM modified row as IVM concentration increases, we chose the EC_50_ of each IVM mediated transition to statisfy $\kappa_{1}<\kappa_{2}<\kappa_{3}$.

The current produced by the model is given by the sum of the currents prodcued by the $\text{open}_{1}$ states $Q_{1}$, $Q_{2}$, $Q_{3}$, and $Q_{4}$ with maximum conductance ($g_{1}$), as well as the $\text{open}_{2}$ states $Q_{5}$, $Q_{6}$, $Q_{7}$, and $Q_{8}$ with conductance ($g_{2}>g_{1}$). As such, it is given by the equation

| $I=g_{1}\left(Q_{1}+Q_{2}+Q_{3}+Q_{4}\right)\left(V-E_{1}\right)+g_{2}\left(Q_{5}+Q_{6}+Q_{7}+Q_{8}\right)\left(V-E_{2}\right)$, | (S1) |
| --- | --- |

where $E_{1}$ ($E_{2}$) is the the reversal potential of $\text{open}_{1}$ ($\text{open}_{2}$) states.

Finally, strong evidence suggests that IVM rescues receptors from an inactivated pool (Fig S5). This is incorporated into the model by the inclusion of the inactivated state $C_{0}$ that exists in a (slow) equilibrium with the closed state $C_{1}$ according to the transition rates $H_{1}$ (from $C_{0}$ to $C_{1}$) and $H_{2}$ (from $C_{1}$ to $C_{0}$). In the presence of IVM, transitions to the IVM modified closed state $C_{3}$ are also possible with a rate $L f_{1}(J)$, where $L$ is the maximum transition rate which is higher than $H_{1}$. Since the simultaneous binding of multiple IVM molecules is unlikely, we only allow for transitions from $C_{0}$ to $C_{1}$ and $C_{3}$.

Based on this discussion and Fig S6, model equations of the one-layer model are thus given by

$$\frac{dC_{0}}{dt}=H_{2}C_{1}+H_{4}Z-\left(H_{1}+Lf_{1}\left(J\right)\right)C_{0}$$

$\frac{dC_{1}}{dt}=H_{1}C_{0}+k_{1}C_{2}+L_{11}C_{3}+k_{r}D_{1}-\left(H_{2}+3k_{2}A+L_{21}f_{1}\left(J\right)\right)C_{1}$ $\frac{dC_{2}}{dt}=3k_{2}AC_{1}+2k_{3}Q_{1}+k_{r}D_{2}+L_{12}C_{4}-\left(k_{1}+3k_{4}A+k_{d}+L_{22}f_{1}\left(J\right)\right)C_{2}$ $\frac{dC_{3}}{dt}=k_{7}C_{4}+Lf_{1}\left(J\right)C_{0}+L_{21}f_{1}\left(J\right)C_{1}+L_{31}C_{5}-\left(3k_{8}A+L_{11}+L_{41}f_{2}\left(J\right)\right)C_{3}$ $\frac{dC_{4}}{dt}=3k_{8}AC_{3}+2k_{9}Q_{3}+L_{22}f_{1}\left(J\right)C_{2}+L_{32}C_{6}-\left(k_{7}+2k_{10}A+L_{12}+L_{42}f_{2}\left(J\right)\right)C_{4}$ $\frac{dC_{5}}{dt}=k_{13}C_{6}+L_{41}f_{2}\left(J\right)C_{3}+L_{51}C_{7}-\left(3k_{14}A+L_{31}+L_{61}f_{3}\left(J\right)\right)C_{5}$ $\frac{dC_{6}}{dt}=3k_{14}AC_{5}+2k_{15}Q_{5}+L_{42}f_{2}\left(J\right)C_{4}+L_{52}C_{8}-\left(k_{13}+2k_{16}A+L_{32}+L_{62}f_{3}\left(J\right)\right)C_{6}$ $\frac{dC_{7}}{dt}=k_{19}C_{8}+L_{61}f_{3}\left(J\right)C_{5}-\left(3k_{20}A+L_{51}\right)C_{7}$ $\frac{dC_{8}}{dt}=3k_{20}AC_{7}+2k_{21}Q_{7}+L_{62}f_{3}\left(J\right)C_{6}-\left(k_{19}+2k_{22}A+L_{52}\right)C_{8}$ $\frac{dQ_{1}}{dt}=2k_{4}AC_{2}+3k_{5}Q_{2}+k_{r}D_{13}+L_{13}Q_{3}-\left(2k_{3}+k_{6}A+k_{d}+L_{23}f_{1}\left(J\right)\right)Q_{1}$ $\frac{dQ_{2}}{dt}=k_{6}AQ_{1}+k_{r}D_{14}+L_{14}Q_{4}-\left(3k_{5}+k_{d}+L_{24}f_{1}\left(J\right)\right)Q_{2}$ $\frac{dQ_{3}}{dt}=2k_{10}AC_{4}+k_{11}Q_{4}+L_{23}f_{1}\left(J\right)Q_{1}+L_{33}Q_{5}-\left(2k_{9}+k_{12}A+L_{13}+L_{43}f_{2}\left(J\right)\right)Q_{3}$ $\frac{dQ_{4}}{dt}=k_{12}AQ_{3}+L_{24}f_{1}\left(J\right)Q_{2}+L_{34}Q_{6}-\left(3k_{11}+L_{14}+L_{44}f_{2}\left(J\right)\right)Q_{4}$ $\frac{dQ_{5}}{dt}=2k_{16}AC_{6}+3k_{17}Q_{6}+L_{43}f_{2}\left(J\right)Q_{3}+L_{53}Q_{7}-\left(2k_{15}+k_{18}A+L_{33}+L_{63}f_{3}\left(J\right)\right)Q_{5}$ $\frac{dQ_{6}}{dt}=k_{18}AQ_{5}+L_{44}f_{2}\left(J\right)Q_{4}+L_{54}Q_{8}-\left(3k_{17}+L_{34}+L_{64}f_{3}\left(J\right)\right)Q_{6}$ $\frac{dQ_{7}}{dt}=2k_{22}AC_{8}+3k_{23}Q_{8}+L_{63}f_{3}\left(J\right)Q_{5}-\left(2k_{21}+k_{24}A+L_{53}\right)Q_{7}$

$$\frac{dQ_{8}}{dt}=k_{24}AQ_{7}+L_{64}f_{3}\left(J\right)Q_{6}-\left(3k_{23}+L_{54}\right)Q_{8}$$

$$\frac{dD_{1}}{dt}=k_{1}D_{1}-\left(3k_{2}A+H_{1}\right)D_{1}$$

$$\frac{dD_{2}}{dt}=3k_{2}AD_{1}+2k_{3}D_{3}+H_{2}C_{2}-\left(k_{1}+2k_{4}A+H_{1}\right)D_{2}$$

$$\frac{dD_{3}}{dt}=2k_{4}AD_{2}+3k_{5}D_{4}+H_{2}Q_{1}-\left(2k_{3}+k_{6}A+H_{1}\right)D_{13}$$

$$\frac{dD_{4}}{dt}=k_{6}AD_{3}+H_{2}Q_{2}-\left(3k_{5}+H_{1}+H_{3}\right)D_{4}$$

$$\frac{dZ}{dt}=H_{3}D_{4}-H_{4}Z$$

**Simulations of P2X4R Gating**

In order to assess the capacity of the one-layer model to reproduce P2X4R gating, we used MCMC techniques (see Methods Section) to determine parameter values that best explain the experimental data. The model was found to be capable of reproducing many of the properties of P2X4R gating. In the absence of IVM, the submodel of the one-layer model (which happens to be identical to the submodel of Zemkova et. al. [1]) was able to capture the experimentally observed naïve receptor activation, desensitization, and deactivation (Fig S7A, F). The increase in both current amplitude (Fig S7B-E), and deactivation time constant (Fig S7F-G) and transient changes in desensitization (Fig S7G) in the presence of IVM were also captured during the pulse protocol, along with the leftward shift in the EC_50_ of the ATP-dependent dose response curves for current amplitude when IVM was applied (Fig S8A).

The decrease in the slope of the I-V curves and the shift in reversal potential generated by a voltage ramp protocol were well accounted for by the model (Fig S8B). We can understand this type of behaviour in terms of Inequalities (4, 6). The previously developed model in [1] was capable of fulfilling these conditions and producing the decrease in the slope of the I-V curves, but it required a large increase (>150%) in unitary conductance to achieve it while simultaneously producing the increase in current amplitude induced by IVM. Since only a 20% increase in unitary conductance has been observed experimentally in P2X4R during IVM application [2], such a large increase in unitary conductance suggested by the model cannot be justified. The one-layer model proposed here kept the increase in unitary conductance within 20% and reproduced the current growth with IVM pretreatment (through IVM-induced transitions from the inactivated state $C_{0}$). Moreover, according to this model, IVM alone was able to tansition receptors to the primed-3 row in the absence of ATP, allowing pretreatment with IVM to produce such effects and to relax the need for a fast priming of receptors necessary for other experiments.

Finally, the one-layer model was successful in reproducing the responses seen in prolonged application of 100 μM ATP in the absence (Fig S8C) and presence of 3 μM IVM (Fig S8D). The former showed partial recovery of peak current response after 3-min ATP washout as seen experimentally. These results suggest that substantial fraction of P2X4R are shielded from desensitization in the presence of IVM (by transitioning receptors to the primed-1, primed-2 and primed-3 rows) and that these four processes exist at equilibrium during the plateauing phase of desensitization.

**Analysis of the model**

The one-layer model possesses a large number of binding parameters. They allow for a very complicated pattern of cooperativity between ATP and IVM binding which manages to reproduce many of the time series of evoked currents very well. With such a large number of states and free parameters, it is valid to question whether the model is simply a machine designed to reproduce the desired output by the MCMC fitting algorithm without being reflective of such cooperativity. The goal of introducing 24 different ATP-associated transition rates was to determine how IVM binding alters cooperativity between ATP binding sites. The Markov model in [1] enforced negative cooperativity in the ATP-binding by ensuring that the affinity of ATP-binding at each step decreases as more ATP molecules bind. To test for cooperativity in the one-layer model, we applied the technique outlined in the Methods Section and found that there was no cooperativity. This model thus suggests that, while IVM does change ATP binding kinetics, it does not alter cooperativity in a manner that is readily discernible from the data.

To further investigate if IVM binding affects the transition rates of ATP binding affinity, we simplified our analysis by employing a similar one-layer model with a smaller number of parameters, where the ATP binding and unbinding rates were substituted by rates that are proportional to those in the naïve row. This was implemented by using the multiplicative factors described in Table S2. Fitting the revised model to the same current recordings used in Fig S7 and S8 revealed that although the number of parameters was reduced from 61 to 49, the overall performance of the model remained roughly the same (results not shown). The values of the multiplicative constants $r_{i}$ and $v_{i}$, $i=1, 2, 3$, obtained from the fitting were: $r_{1}=3.09$, $r_{2}=3.05$, $r_{3}=2.61$ and $v_{1}=0.17$, $v_{2}=0.035$, $v_{3}=0.0075$, which seemed to suggest that IVM binding successively decreases the unbinding rate of ATP to P2X4R (i.e., increases the binding affinity of ATP). The larger number of degrees of freedom in the one-layer model and their compensatory nature prohibited fruitful analysis of the model prior to simplification with multiplicative factors, yet it allowed for a significant increase in the capacity of the model to simultaneously capture the complex experimental behaviour of different cells.

**Appendix B**

**Description of the Two-Layer Model**

While the one-layer model had many of the necessary ingredients to capture the gating properties of P2X4R and their allosteric modulation by IVM, there was a rather obvious issue with the one-layer model tested. Generally speaking, it did not robustly capture the short timescales of activation and desensitization; it did so by keeping a delicate balance between IVM binding rates which was unnecessary. This type of behaviour is a consequence of the fact that the one-layer model does not allow for IVM bound receptors to desensitize. It only allows receptors in the naïve row to do so which means that IVM-bound receptors must first lose all bound IVM molucles to desensitize. This is at odds with our findings about the transient increase in desensitization rate (captured by the model through having IVM unbinding rates increase with ATP binding) and with the general function of P2X4R at high IVM concentrations (where receptors desensitize but, according to the one-layer model, they are concentrated in the primed-1, primed-2 and primed-3 rows).

Much of this work was motivated by the inability of the one-layer model to faithfully reproduce the short timescale of receptor activation and desensitization. Based on this model, IVM binding creates a functional stage from copies of an ATP binding row without copying its desensitized states. Since P2X4R appear to desensitize even at high IVM concentrations, we concluded that IVM binding should also create copies of its desensitized states as suggested by the gating scheme of Fig 2A. We have therefore revised the one-layer model by assuming that IVM bound (closed and open) states of P2X4R can desensitize. We termed this model, the two-layer model, where the upper layer (the functional stage) is identical to the one-layer model, without the desensitized row, and the lower layer (the desensitized stage) is comprised of only desensitized states possessing the same transition rates as the upper layer. Transitions from the upper layer to the lower layer of the model represent receptor desensitization and is denoted by $k_{d,i}$, $i=1, 2, 3, 4$, whereas transitions from the lower layer to the upper layer represent receptor recovery and is denoted by $k_{r,i}$, $i=1, 2, 3, 4$. The remaining transitions are identical to those seen in the one-layer model. Despite their differences in desensitization kinetics, the current produced by the two-layer model also obeys Eq. (S1) associated with the one-layer model.

Model equations of the two-layer model, as described by Fig 3, are thus given by

$$\frac{dC_{0}}{dt}=H_{2}C_{1}-\left(H_{1}+Lf_{1}\left(J\right)\right)C_{0}$$

$\frac{dC_{1}}{dt}=H_{1}C_{0}+k_{1}C_{2}+L_{11}C_{3}+k_{r,1}D_{11}+H_{4}Z-\left(H_{2}+3k_{2}A+L_{21}f_{1}\left(J\right)\right)C_{1}$ $\frac{dC_{2}}{dt}=3k_{2}AC_{1}+2k_{3}Q_{1}+k_{r,1}D_{12}+L_{12}C_{4}-\left(k_{1}+2k_{4}A+k_{d,1}+L_{22}f_{1}\left(J\right)\right)C_{2}$

$$\frac{dC_{3}}{dt}=k_{7}C_{4}+Lf_{1}\left(J\right)C_{0}+L_{21}f_{1}\left(J\right)C_{1}+L_{31}C_{5}+k_{r,2}D_{21}-\left(3k_{8}A+L_{11}+L_{41}f_{2}\left(J\right)\right)C_{3}$$

$\frac{dC_{4}}{dt}=3k_{8}AC_{3}+2k_{9}Q_{3}+k_{r,2}D_{22}+L_{22}f_{1}\left(J\right)C_{2}+L_{32}C_{6}-\left(k_{7}+2k_{10}A+k_{d,2}+L_{12}+L_{42}f_{2}\left(J\right)\right)C_{4}$ $\frac{dC_{5}}{dt}=k_{13}C_{6}+L_{41}f_{2}\left(J\right)C_{3}+L_{51}C_{7}+k_{r,3}D_{31}-\left(3k_{14}A+L_{31}+L_{61}f_{3}\left(J\right)\right)C_{5}$

$\frac{dC_{6}}{dt}=3k_{14}AC_{5}+2k_{15}Q_{5}+k_{r,3}D_{32}+L_{42}f_{2}\left(J\right)C_{4}+L_{52}C_{8}-\left(k_{13}+2k_{16}A+k_{d,3}+L_{32}+L_{62}f_{3}\left(J\right)\right)C_{6}$ $\frac{dC_{7}}{dt}=k_{19}C_{8}+L_{61}f_{3}\left(J\right)C_{5}+k_{r,4}D_{41}-\left(3k_{20}A+L_{51}\right)C_{7}$

$$\frac{dC_{8}}{dt}=3k_{20}AC_{7}+2k_{21}Q_{7}+k_{r,4}D_{42}+L_{62}f_{3}\left(J\right)C_{6}-\left(k_{19}+2k_{22}A+k_{d,4}+L_{52}\right)C_{8}$$

$$\frac{dQ_{1}}{dt}=2k_{4}AC_{2}+3k_{5}Q_{2}+k_{r,1}D_{13}+L_{13}Q_{3}-\left(2k_{3}+k_{6}A+k_{d,1}+L_{23}f_{1}\left(J\right)\right)Q_{1}$$

$\frac{dQ_{2}}{dt}=k_{6}AQ_{1}+k_{r,1}D_{14}+L_{14}Q_{4}-\left(3k_{5}+k_{d,1}+L_{24}f_{1}\left(J\right)\right)Q_{2}$ $\frac{dQ_{3}}{dt}=2k_{10}AC_{4}+3k_{11}Q_{4}+k_{r,2}D_{23}+L_{23}f_{1}\left(J\right)Q_{1}+L_{33}Q_{5}-\left(2k_{9}+k_{12}A+k_{d,2}+L_{13}+L_{43}f_{2}\left(J\right)\right)Q_{3}$ $\frac{dQ_{4}}{dt}=k_{12}AQ_{3}+k_{r,2}D_{24}+L_{24}f_{1}\left(J\right)Q_{2}+L_{34}Q_{6}-\left(3k_{11}+k_{d,2}+L_{14}+L_{44}f_{2}\left(J\right)\right)Q_{4}$ $\frac{dQ_{5}}{dt}=2k_{16}AC_{6}+3k_{17}Q_{6}+k_{r,3}D_{33}+L_{43}f_{2}\left(J\right)Q_{3}+L_{53}Q_{7}-\left(2k_{15}+k_{18}A+k_{d,3}+L_{33}+L_{63}f_{3}\left(J\right)\right)Q_{5}$ $\frac{dQ_{6}}{dt}=k_{18}AQ_{5}+k_{r,3}D_{34}+L_{44}f_{2}\left(J\right)Q_{4}+L_{54}Q_{8}-\left(3k_{17}+k_{d,3}+L_{34}+L_{64}f_{3}\left(J\right)\right)Q_{6}$ $\frac{dQ_{7}}{dt}=2k_{22}AC_{8}+3k_{23}Q_{8}+k_{r,4}D_{43}+L_{63}f_{3}\left(J\right)Q_{5}-\left(2k_{21}+k_{24}A+k_{d,4}+L_{53}\right)Q_{7}$

$$\frac{dQ_{8}}{dt}=k_{24}AQ_{7}+k_{r,4}D_{44}+L_{64}f_{3}\left(J\right)Q_{6}-\left(3k_{23}+k_{d,4}+L_{54}\right)Q_{8}$$

$$\frac{dD_{11}}{dt}=k_{1}D_{12}+L_{11}D_{21}-\left(3k_{2}A+L_{21}f_{1}\left(J\right)+k_{r,1}\right)D_{11}$$

$$\frac{dD_{12}}{dt}=3k_{2}AD_{11}+2k_{3}D_{13}+k_{d,1}C_{2}+L_{12}D_{22}-\left(k_{1}+2k_{4}A+k_{r,1}+L_{22}f_{1}\left(J\right)\right)D_{12}$$

$$\frac{dD_{13}}{dt}=2k_{4}AD_{12}+3k_{5}D_{14}+k_{d,1}Q_{1}+L_{13}D_{23}-\left(2k_{3}+k_{6}A+k_{r,1}+L_{23}f_{1}\left(J\right)\right)D_{13}$$

$\frac{dD_{14}}{dt}=k_{6}AD_{13}+k_{d,1}Q_{2}+L_{14}D_{24}-\left(3k_{5}+k_{r,1}+L_{24}f_{1}\left(J\right)+H_{3}\right)D_{14}$ $\frac{dD_{21}}{dt}=k_{7}D_{22}+L_{21}f_{1}\left(J\right)D_{11}+L_{31}D_{32}-\left(3k_{8}A+L_{11}+L_{41}f_{2}\left(J\right)+k_{r,2}\right)D_{21}$ $\frac{dD_{22}}{dt}=3k_{8}AD_{21}+2k_{9}D_{23}+k_{d,2}C_{4}+L_{22}f_{1}\left(J\right)D_{12}+L_{32}D_{31}-\left(k_{7}+2k_{10}A+k_{r,2}+L_{12}+L_{42}f_{2}\left(J\right)\right)D_{22}$ $\frac{dD_{23}}{dt}=2k_{10}AD_{22}+3k_{11}D_{24}+k_{d,2}Q_{3}+L_{23}f_{1}\left(J\right)D_{13}+L_{33}D_{33}-\left(2k_{9}+k_{12}A+k_{r,2}+L_{13}+L_{43}f_{2}\left(J\right)\right)D_{23}$ $\frac{dD_{24}}{dt}=k_{12}AD_{23}+k_{d,2}Q_{4}+L_{24}f_{1}\left(J\right)D_{14}+L_{34}D_{34}-\left(3k_{11}+k_{r,2}+L_{14}+L_{44}f_{2}\left(J\right)+H_{5}\right)D_{24}$

$$\frac{dD_{31}}{dt}=k_{13}D_{32}+L_{42}f_{2}\left(J\right)D_{22}+L_{51}D_{41}-\left(3k_{14}A+L_{32}+L_{61}f_{3}\left(J\right)+k_{r,3}\right)D_{31}$$

$\frac{dD_{32}}{dt}=3k_{14}AD_{31}+2k_{15}D_{33}+k_{d,3}C_{6}+L_{41}f_{2}\left(J\right)D_{21}+L_{52}D_{42}-\left(k_{13}+2k_{16}A+k_{r,3}+L_{31}+L_{62}f_{3}\left(J\right)\right)D_{32}$ $\frac{dD_{33}}{dt}=2k_{16}AD_{32}+3k_{17}D_{34}+k_{d,3}Q_{5}+L_{43}f_{2}\left(J\right)D_{23}+L_{53}D_{43}-\left(2k_{15}+k_{18}A+k_{r,3}+L_{33}+L_{63}f_{3}\left(J\right)\right)D_{33}$ $\frac{dD_{34}}{dt}=k_{18}AD_{33}+k_{d,3}Q_{6}+L_{44}f_{2}\left(J\right)D_{24}+L_{54}D_{44}-\left(3k_{17}+k_{r,3}+L_{34}+L_{64}f_{3}\left(J\right)+H_{5}\right)D_{34}$

$$\frac{dD_{41}}{dt}=k_{19}D_{42}+L_{61}f_{3}\left(J\right)D_{31}-\left(3k_{20}A+L_{51}+k_{r,4}\right)D_{41}$$

$$\frac{dD_{42}}{dt}=3k_{20}AD_{41}+2k_{21}D_{43}+k_{d,4}C_{8}+L_{62}f_{3}\left(J\right)D_{32}-\left(k_{19}+2k_{22}A+k_{r,4}+L_{52}\right)D_{42}$$

$$\frac{dD_{43}}{dt}=2k_{22}AD_{42}+3k_{23}D_{44}+k_{d,4}Q_{7}+L_{63}f_{3}\left(J\right)D_{33}-\left(2k_{21}+k_{24}A+k_{r,4}+L_{53}\right)D_{43}$$

$$\frac{dD_{44}}{dt}=k_{24}AD_{43}+k_{d,4}Q_{8}+L_{64}f_{3}\left(J\right)D_{34}-\left(3k_{23}+k_{r,4}+L_{54}+H_{5}\right)D_{44}$$

$\frac{dZ}{dt}=H_{3}D_{14}+H_{5}D_{24}+H_{5}D_{34}+H_{5}D_{44}-H_{4}Z$.

Detailed analysis of the model and the results are presented in the main text.

**Appendix C**

**Determination of Model Reversal Potentials**

Here we derive the expressions used to determine the reversal potential of the $\text{open}_{1}$states $Q_{1}$, $Q_{2}$, $Q_{3}$, and $Q_{4}$ ($E_{1}$) and the $\text{open}_{2}$ states $Q_{5}$, $Q_{6}$, $Q_{7}$, and $Q_{8}$ ($E_{2}$) in asymmetric ionic conditions (NMDG^+^ outside, Na^+^ inside) during parameter fitting. The current Eq. (S1) contains the two reversal potentials $E_{1}$ and $E_{2}$. Under symmertic ionic conditions (Fig 4 and Fig 5A, C, D), they are both assumed to be zero. However, in the presence of IVM and in asymmetric conditions, P2X4Rs display altered selectivity (permeability) towards NMDG^+^ which results in shift in the reversal potential over time (see Results). To capture this phenomena, we will assume that the $\text{open}_{1}$ and $\text{open}_{2}$ states differ in their reversal potentials in NMDG^+^ containing media. Given the complex time-dependent relationship between ionic conditions, channel permeability, and reversal potential, it is important to note that the reversal potential of the $\text{open}_{2}$ state $E_{2}$ reflects not only the change in selectivity of chanel pore to NMDG^+^, but also the depletion of intracellular Na^+^ and accumulation of NMDG^+^ inside the cell as has been suggested in [3]. As a result, we only show the first and last I-V curves produced by the ramp protocol to demonstrate the shift (see Fig 5B and Fig S8B).

It it possible to explicitly determine the reversal potentials $E_{1}$ and $E_{2}$ in NMDG^+^ containing media using Eq. (S1) and the voltage ramp data of Fig S1 (along with its statistics presented in [1]). According to Eq. (1), we can rewrite Eq. (S1) as follows

$I\left(t\right)=g_{1}P\left(\text{open}_{ 1}|t\right) \left(V-E_{1}\right)+g_{2}P\left(\text{open}_{ 2}|t\right) \left(V-E_{2}\right)$,

where $P\left(\text{open}_{ 1}|t\right)$ and $P\left(\text{open}_{ 2}|t\right)$ are the probabilities of being in the conductive pore states $\text{open}_{1}$ and $\text{open}_{2}$ at time $t$, respectively. For a given set of model kinetic parameters, $x=(p_{1},p_{2},\ldots,p_{N})$, it is possible to determine these probabilities at all points in time by numerical simulation.

As was done in the Results section, we can rewrite the previous current equation as

$I\left(t\right)=g_{tot}\left(t\right) \left(V-E_{tot}\left(t\right)\right)$,

where $g_{tot}\left(t\right)$ and $E_{tot}\left(t\right)$ are the time-dependent total conductance and reversal potential, resepectively. Based on the description above, we conclude that the total reversal potential is given by

$E_{tot}\left(t\right)=\frac{g_{1}P\left(\text{open}_{ 1}|t\right)E_{1}+g_{2}P\left(\text{open}_{ 2}|t\right)E_{2}}{g_{1}P\left(\text{open}_{ 1}|t\right)+g_{2}P\left(\text{open}_{ 2}|t\right)}$.

Given $E_{tot}\left(t_{i}\right)$ and $E_{tot}\left(t_{f}\right)$, the experimentally determined reversal potentials at the beginning (-38.2 mV) and at the end (-27.4 mV) of the ATP application in the ramp protocol [1], respectively, and given $P\left(\text{open}_{ 1}\right)$ and $P\left(\text{open}_{ 2}\right)$, the numerically determined values of the open probabilities at $t_{i}$ and $t_{f}$ for a given set of kinetic parameter values, $x$, one can determine the values of $E_{1}$ and $E_{2}$ as follows

| $\begin{matrix} E_{1}\left(x\right)=\frac{E_{tot}\left(t_{f}\right)P\left(\text{open}_{ 1}\vert t_{f},x\right)P\left(\text{open}_{ 2}\vert t_{i},x\right)-E_{tot}\left(t_{i}\right)P\left(\text{open}_{ 1}\vert t_{i},x\right)P\left(\text{open}_{ 2}\vert t_{f},x\right)}{\left(P\left(\text{open}_{1}\vert t_{i},x\right)P\left(\text{open}_{ 2}\vert t_{f},x\right)-P\left(\text{open}_{ 1}\vert t_{f},x\right)P\left(\text{open}_{ 2}\vert t_{i},x\right)\right)} \\ +\frac{g_{2}\left[E_{tot}\left(t_{f}\right)-E_{tot}\left(t_{i}\right)\right]P\left(\text{open}_{ 2}\vert t_{i},x\right)P\left(\text{open}_{ 2}\vert t_{f},x\right)}{g_{1}\left(P\left(\text{open}_{ 1}\vert t_{i},x\right)P\left(\text{open}_{ 2}\vert t_{f},x\right)-P\left(\text{open}_{ 1}\vert t_{f},x\right)P\left(\text{open}_{ 2}\vert t_{i},x\right)\right)} \end{matrix}$ | (S2) |
| --- | --- |

and

| $\begin{matrix} E_{2}\left(x\right)=\frac{E_{tot}\left(t_{f}\right)P\left(\text{open}_{ 1}\vert t_{i},x\right)P\left(\text{open}_{ 2}\vert t_{f},x\right)-E_{tot}\left(t_{i}\right)P\left(\text{open}_{ 1}\vert t_{f},x\right)P\left(\text{open}_{ 2}\vert t_{i},x\right)}{\left(P\left(\text{open}_{ 1}\vert t_{i},\theta\right)P\left(\text{open}_{ 2}\vert t_{f},\theta\right)-P\left(\text{open}_{ 1}\vert t_{f},\theta\right)P\left(\text{open}_{ 2}\vert t_{i},\theta\right)\right)} \\ +\frac{g_{1}\left[E_{tot}\left(t_{f}\right)-E_{tot}\left(t_{i}\right)\right]P\left(\text{open}_{ 1}\vert t_{0},x\right)P\left(\text{open}_{ 1}\vert t_{f},x\right)}{g_{2}\left(P\left(\text{open}_{ 1}\vert t_{i},x\right)P\left(\text{open}_{ 2}\vert t_{f},x\right)-P\left(\text{open}_{ 1}\vert t_{f},x\right)P\left(\text{open}_{ 2}\vert t_{i},x\right)\right)}. \end{matrix}$ | (S3) |
| --- | --- |

As was previously demonstrated in [3] and as suggested by the Goldman-Hodgkin-Katz equation, ionic exchanges across the membrane, when the channels are conducting (i.e., when they are in the $\text{open}_{ 1}$ and $\text{open}_{ 2}$ states), contribute significantly to the observed shift in reversal potential. Because we do not explicitly model flux of ionic species across the cellular membrane, the value of $E_{1}$ determined by the procedure described above is unlikely to correspond to the reversal potential of the pore in the absence of IVM. Nonetheless, the values of $E_{1}$ and $E_{2}$ will reproduce the experimentally observed shift in reversal potential with high accuracy.

**References**

1. Zemkova, H., et al., *Allosteric regulation of the P2X4 receptor channel pore dilation.* Pflügers Archiv-European Journal of Physiology, 2015. **467**(4): p. 713-726.

2. Priel, A. and S.D. Silberberg, *Mechanism of ivermectin facilitation of human P2X4 receptor channels.* The Journal of general physiology, 2004. **123**(3): p. 281-293.

3. Li, M., et al., *Physical basis of apparent pore dilation of ATP-activated P2X receptor channels.* Nature neuroscience, 2015.
