# Supplementary figures and images for: Deciphering the regulation of P2X4 receptor channel gating by ivermectin using Markov models
Source: PLoS Comput Biol. 2017 Jul 14;13(7):e1005643. doi: 10.1371/journal.pcbi.1005643 (PMC5533465; doi:10.1371/journal.pcbi.1005643)

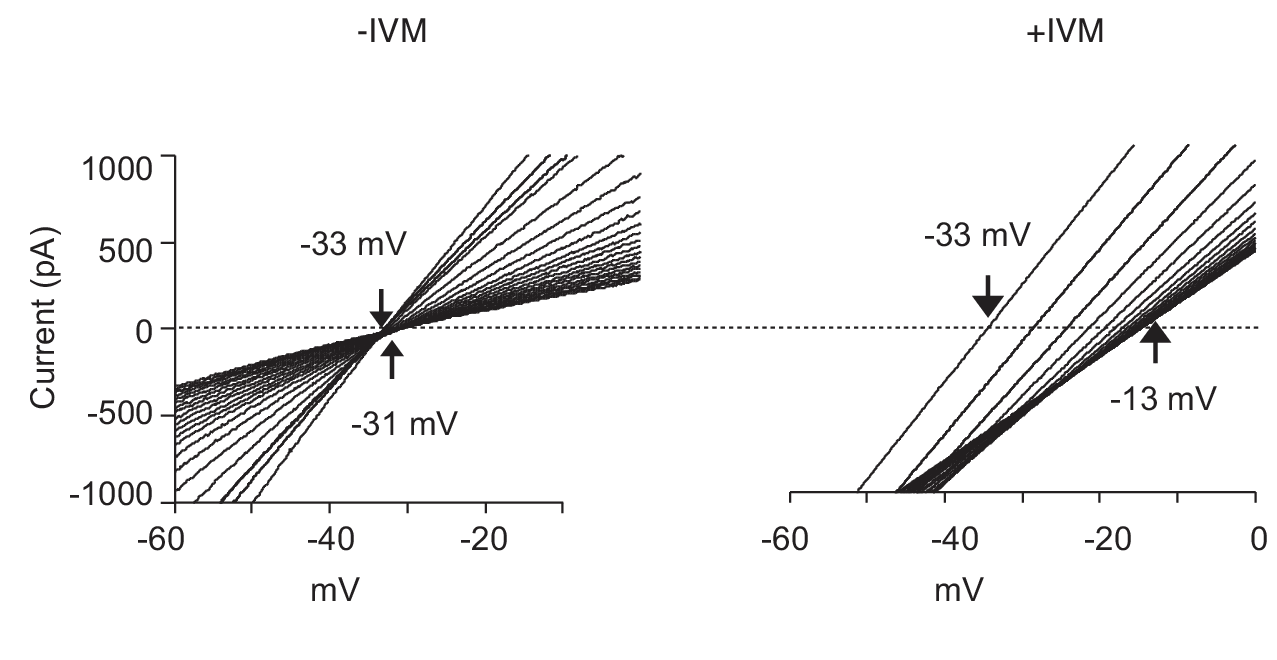

Supplement: S1 Fig — Cells were bathed in extracellular solution containing: 155 mM NMDG+, 3 mM KCl, 2 mM CaCl2, 1 mM MgCl2, 10 mM HEPES and 10 mM D-glucose, adjusted to pH 7.3 with HCl. Patch electrodes were filled with solution containing 145 mM NaCl, 10 mM EGTA, and 10 mM HEPES. I-V curves showing how IVM shifts reversal potential during voltage ramp experiments. Substituting Na+ by NMDG+ in the medium does not change reversal potential in the absence of IVM (left) but shifts it from −33 mV to −13 mV in cells pretreated with 3 μM IVM for 30–60 s (right). In both cases, cells are stimulated with 100 μM ATP for 10 s and the voltage is ramped from −80 to +80 mV twice per second. A decrease in the total conductance of P2X4R (manifested as a decrease in the slope of the I-V curves) is observed under both conditions. Traces shown are representative of 30 similar experiments. Figure adapted with permission from [20]. (TIF) [file pcbi.1005643.s002.tif]

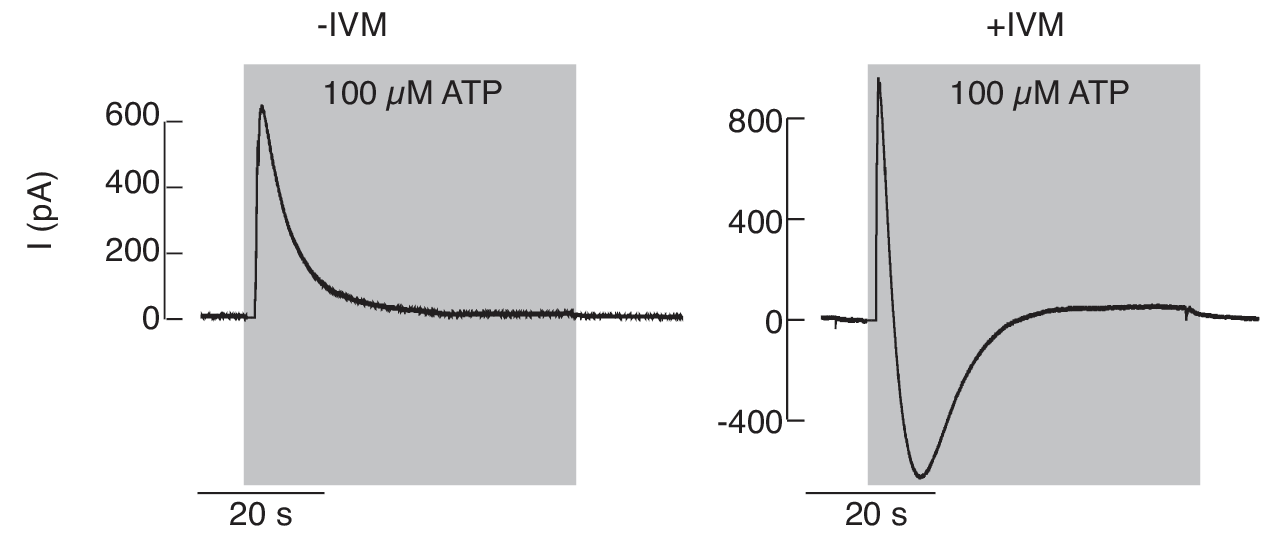

Supplement: S2 Fig — Currents induced by 100 μM ATP were recorded at a −60-mV holding potential during 60-s agonist application in the absence (left) and presence of IVM (right). In contrast to the recordings in S1 Fig (which were performed in the presence of Ca2+), the recordings presented here were performed in the absence of extracellular Ca2+, making the reversal potential for ATP-induced current more negative (about −70 mV [27]) than that in S1 Fig. (TIF) [file pcbi.1005643.s003.tif]

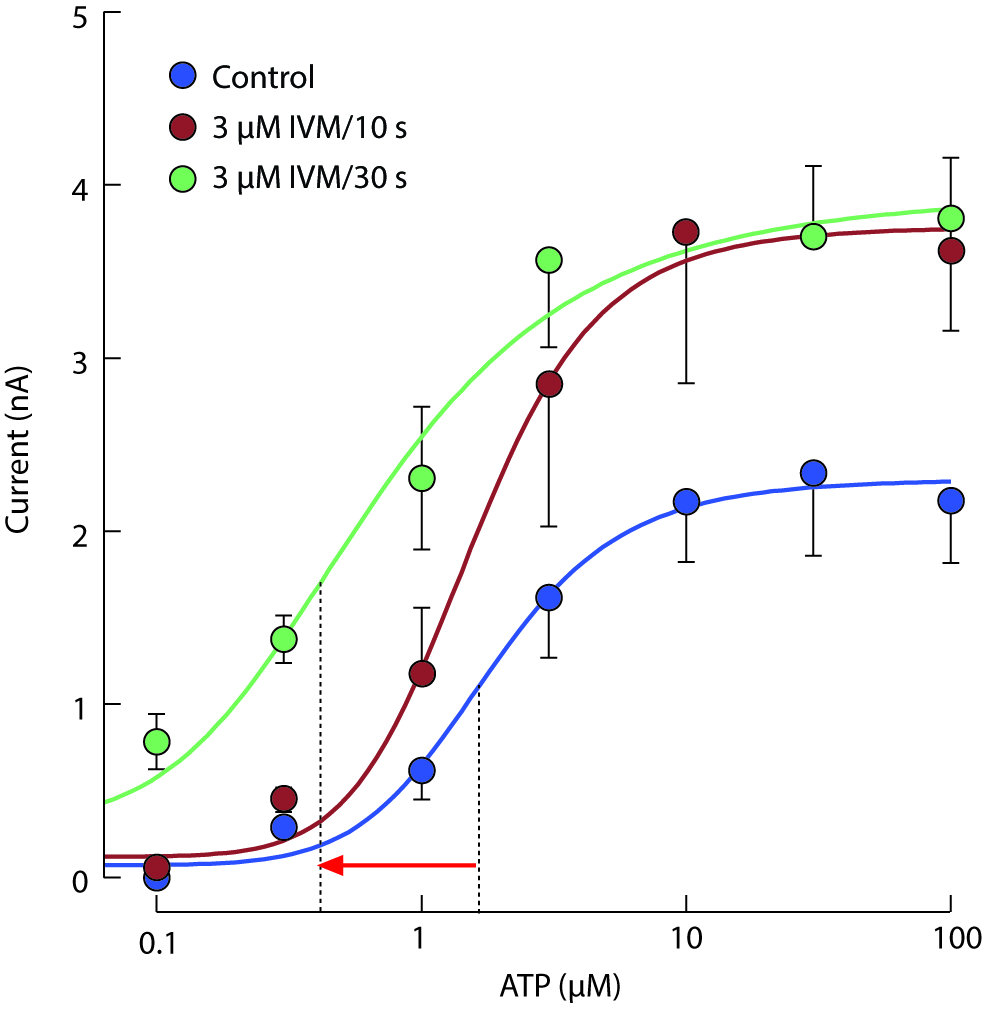

Supplement: S3 Fig — The cells were pretreated with IVM for 10 s (maroon circles) or for 30 s (green circles). Red arrow indicates the magnitude of the shift in the EC50. Data shown are mean±SEM values from n = 4–20 cells per dose. Figure adapted with permission from [20]. (TIF) [file pcbi.1005643.s004.tif]

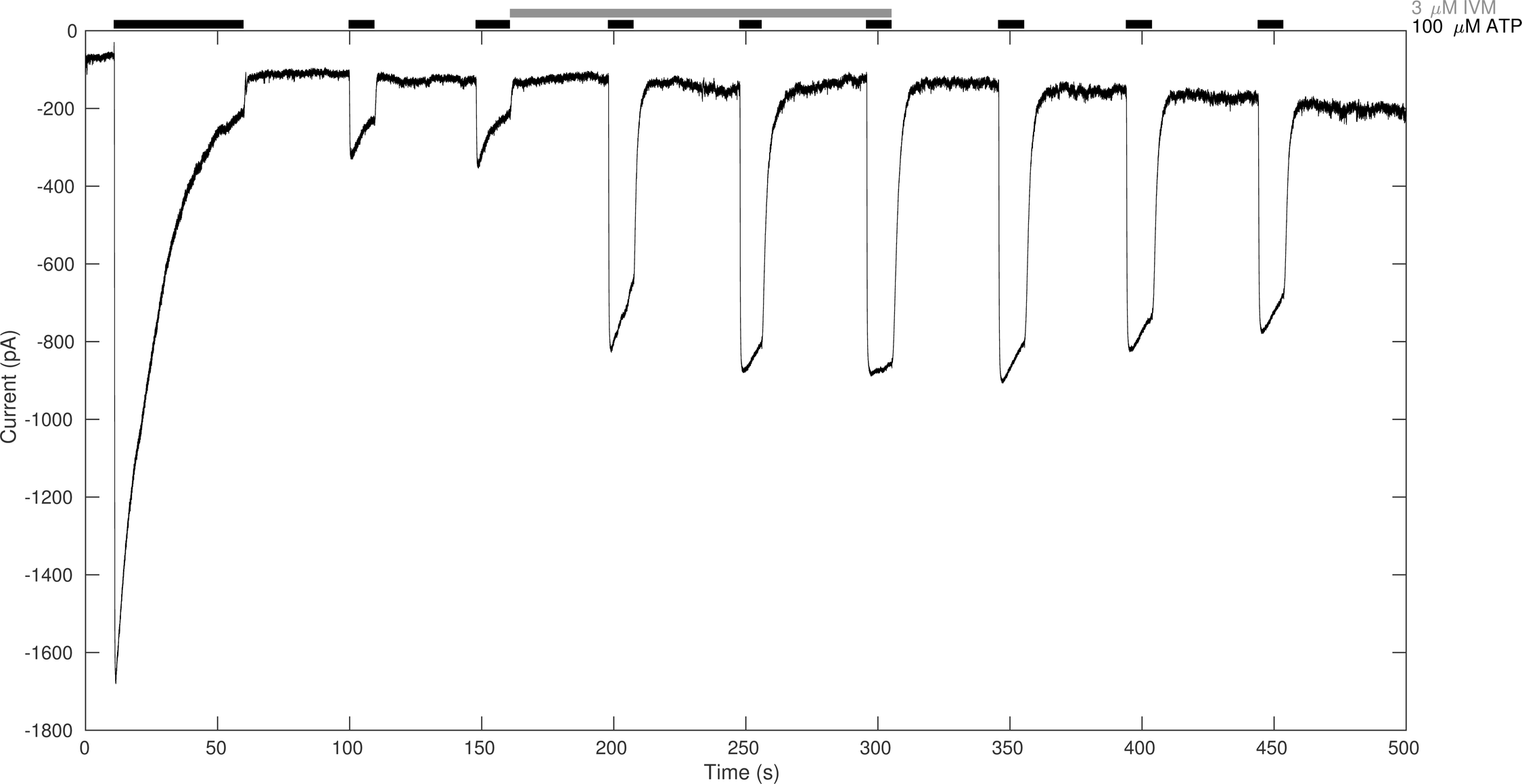

Supplement: S4 Fig — A 50-s application with 100 μM ATP is used to desensitize nearly all P2X4R, followed by two 10-s ATP applications separated by 40-s washout periods demonstrating minimal recovery during washout. The subsequent addition of 3 μM IVM extracellularly in combination with 10-s ATP applications reveal an increase in receptor activation that persists even after IVM is removed 145 s later. (TIF) [file pcbi.1005643.s005.tif]

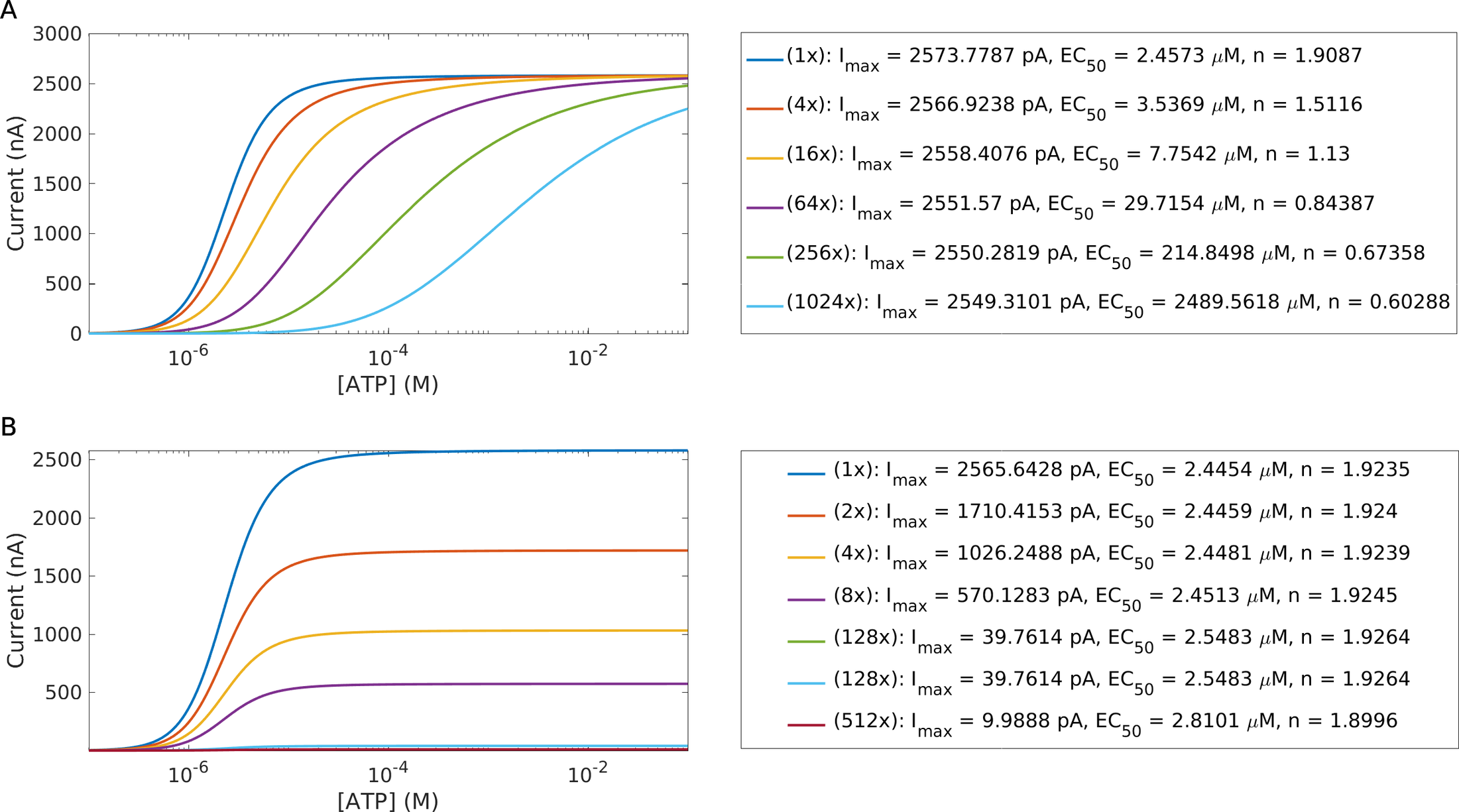

Supplement: S5 Fig — Hill functions were fit to ATP-dependent concentration-response curves of peak current amplitude generated using the gating schemes in Fig 2A and 2B, respectively. Enhanced desensitization and inactivation were realized by altering the magnitude of kd in A and H1 in B, respectively, by a factor indicated in the legends. The fitted parameters of each Hill function (including Imax, EC50 and n) for both cases are also presented in the legends. Enhanced desensitization increases EC50 and n, whereas enhanced inactivation decreases Imax. Note that the range of agonist concentration along the x-axis spans 6 orders of magnitude but all responses in B reach saturation within 2 orders of magnitude, as observed experimentally (compare to S3 Fig), unlike in panel A. (TIF) [file pcbi.1005643.s006.tif]

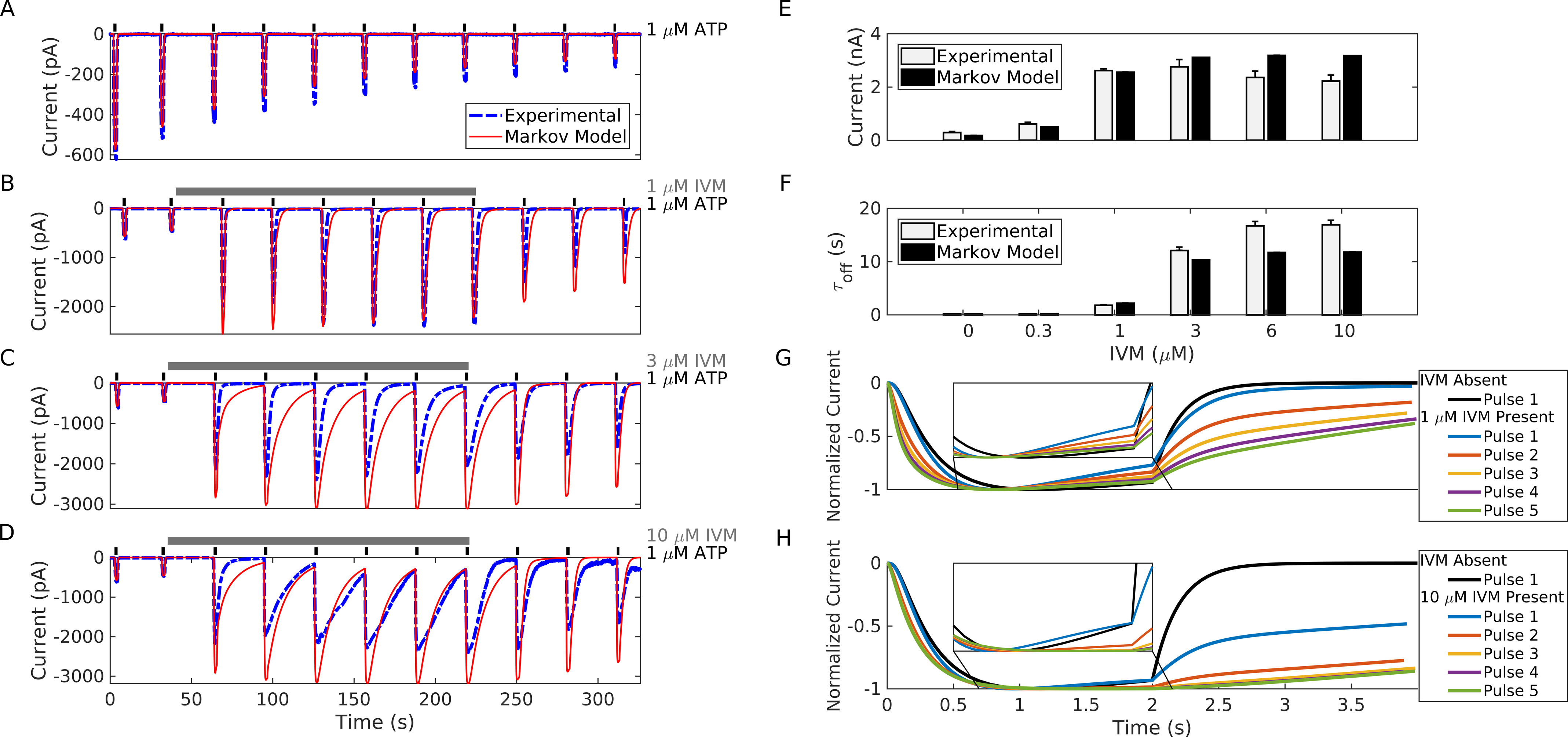

Supplement: S7 Fig — (A-D) Time series of the current in the pulse protocol performed with (A) 0, (B) 1, (C) 3, and (D) 10 μM IVM. Dashed blue lines, experimental recordings; solid red lines, model simulations. (E, F) IVM-dependent concentration-response curves of (E) peak current amplitude and (F) rate of receptor deactivation. Model deactivation kinetics τoff are measured by the weighted time constant. (G, H) Progression of activation, desensitization, and deactivation of currents produced by the model when normalized by maximum amplitudes during the pulse protocol in the presence of 1 μM (G) and 10 μM (H) IVM. All experimental data are derived from [20]. (TIF) [file pcbi.1005643.s008.tif]

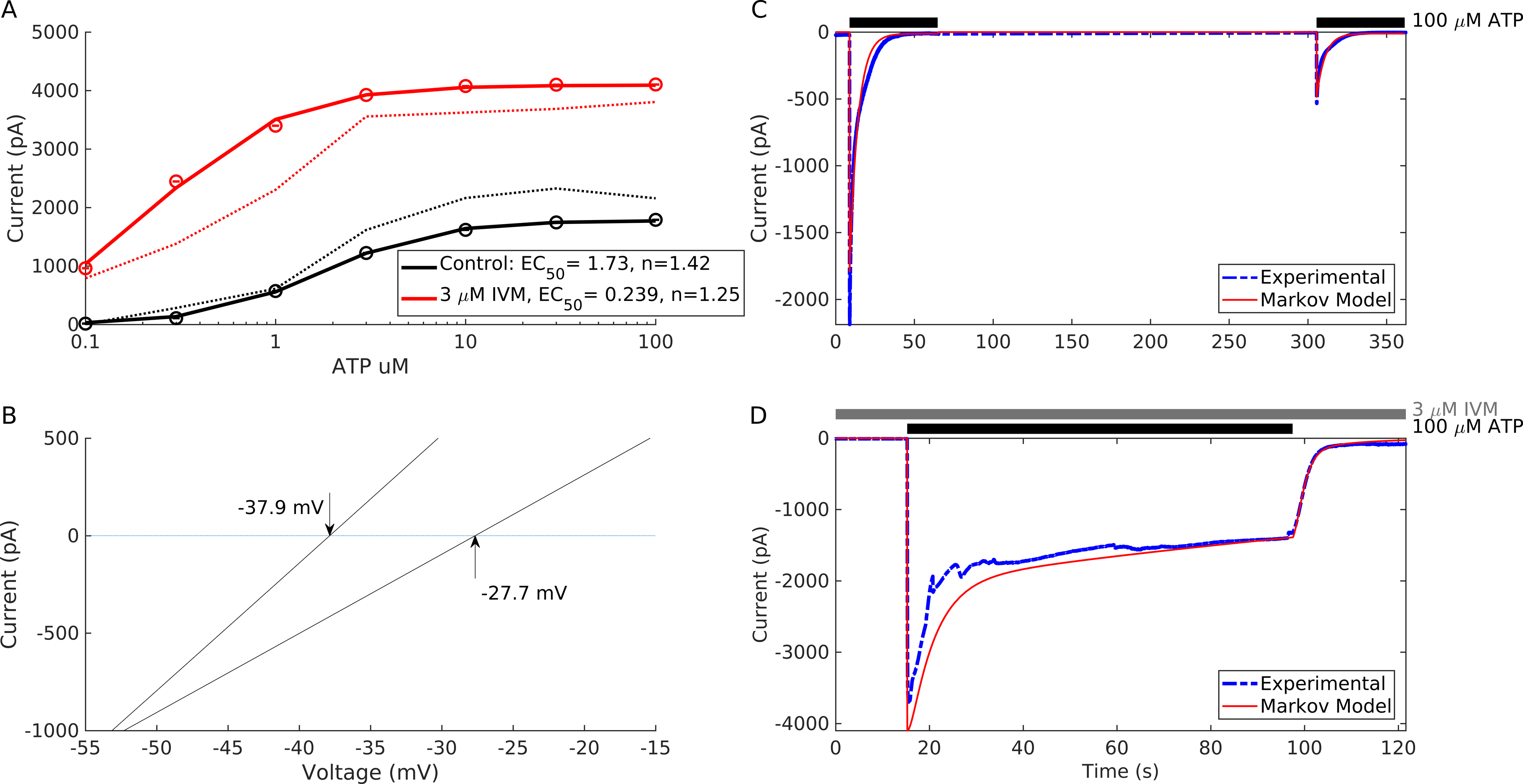

Supplement: S8 Fig — (A) ATP-dependent dose-response curves in the absence (black) and presence (red) of 3 μM IVM applied 30 s before ATP stimulation. Calculated EC50 and Hill coefficients in the absence of IVM were 1.7306 μM and 1.4244, respectively, and in the presence of IVM were 0.2386 μM and 1.2465, respectively. Dotted lines are experimental data. (B) The first and last I-V curves showing the decrease in their slopes and a positive shift in reversal potential upon stimulation with 100 μM ATP for 10 s in the presence of 3 μM IVM applied 20 s before ATP stimulation. The bath medium had Na+ replaced by NMDG+ modeled by setting E1 = −46.1 mV and E2 = −21.9 mV. The voltage is ramped from −80 mV to +80 mV twice per second from a holding potential of −60 mV. (C), Two prolonged applications of 100 μM ATP produced by the model in the absence of IVM separated by a 3 min washout period. (D) Prolonged application of 100 μM ATP produced by the model in the presence of 3 μM IVM. All experimental data are derived from [20]. (TIF) [file pcbi.1005643.s009.tif]

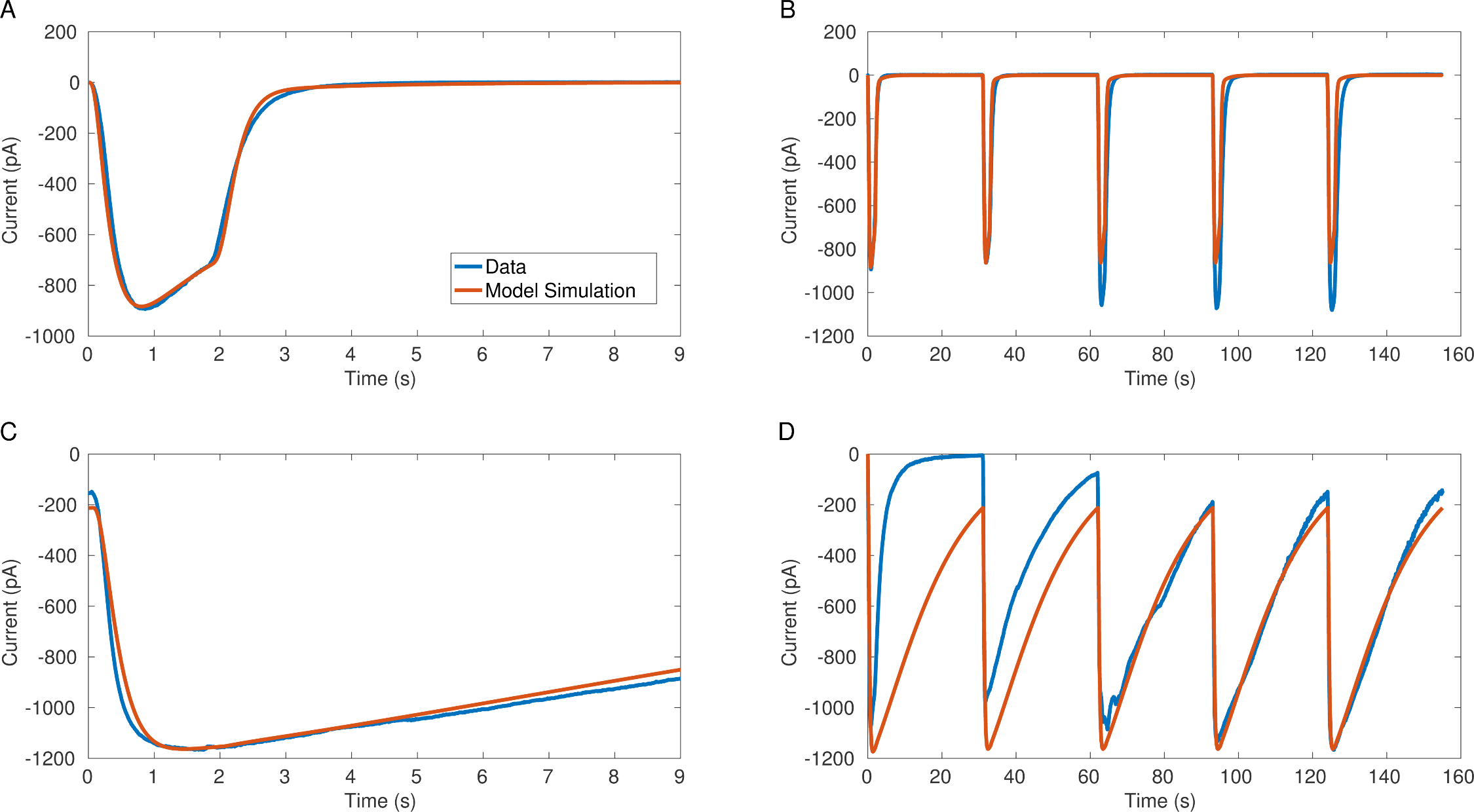

Supplement: S9 Fig — (A) Single gating scheme fitting (orange line) to the first pulse (before IVM application) of a 1 μM IVM pulse protocol recording (blue line) derived from experiments shown in S7B Fig. (B) The remainder of the current time series produced by the gating scheme used to fit the pulse in A. (C) Single gating scheme fitting (orange line) to the last pulse (before IVM washout) of a 10 μM IVM pulse protocol recording (blue line) derived from experiments shown in S7D Fig. (D) The remainder of the current time series produced by the gating scheme used to fit the pulse in (C). All experimental data are derived from [20]. (TIF) [file pcbi.1005643.s010.tif]

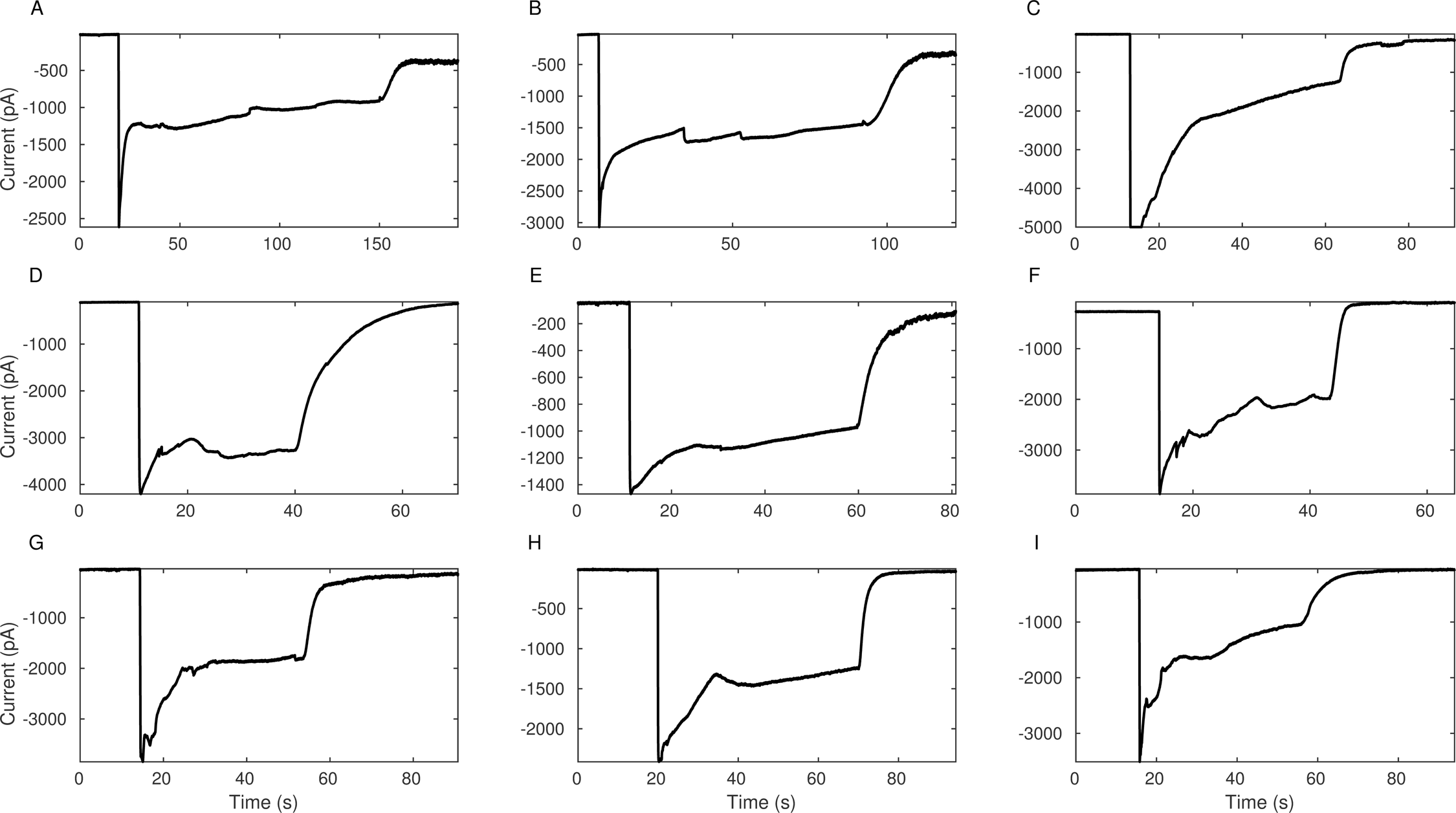

Supplement: S10 Fig — Experimental conditions are identical to those used to produce the data in Fig 5D. (TIF) [file pcbi.1005643.s011.tif]

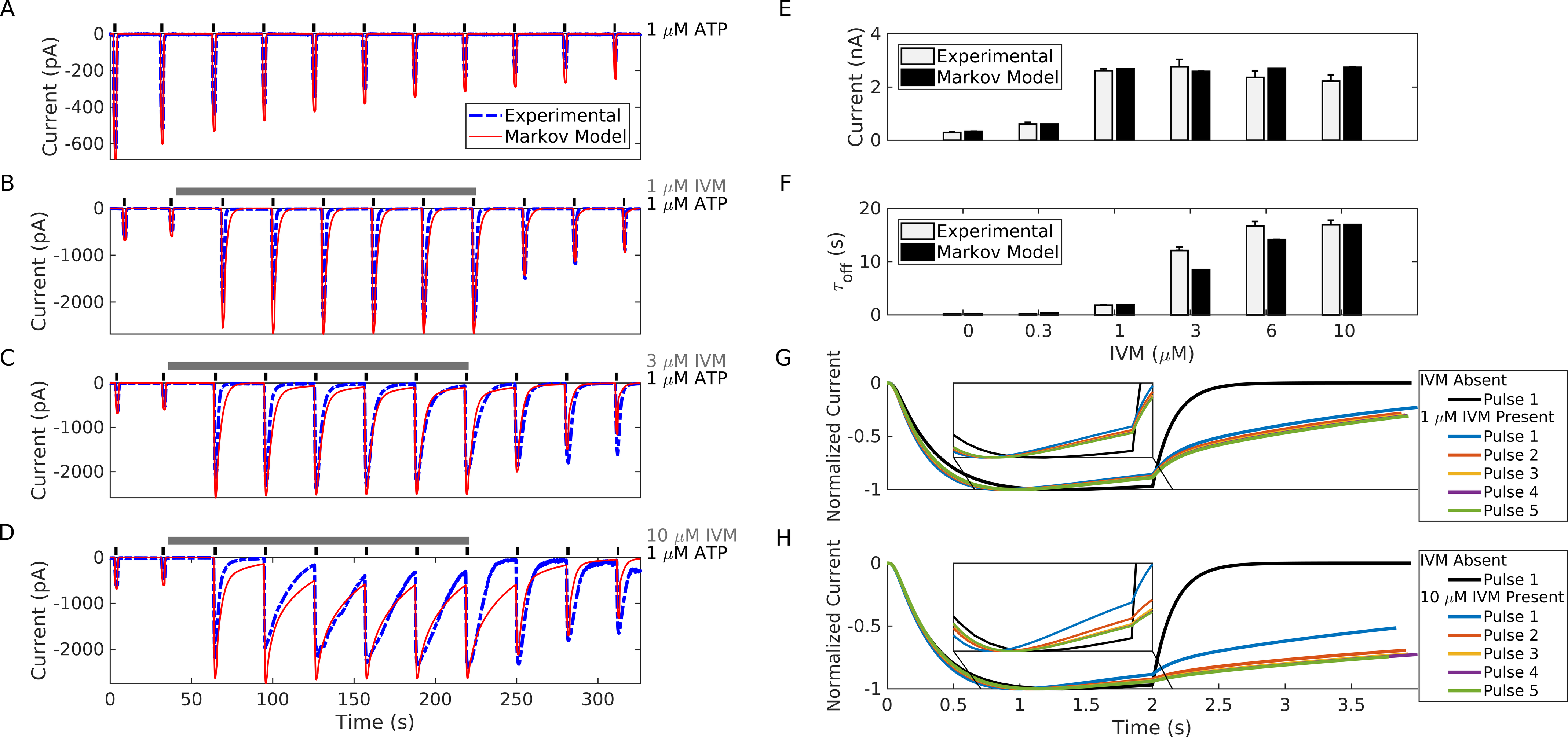

Supplement: S11 Fig — (A-D) Simulated time series of current for the pulse protocol performed with (A) 0, (B) 1, (C) 3, and (D) 10 μM IVM. Dashed blue lines, experimental recordings; solid red lines, simulations. (E, F) IVM-dependent concentration-response curves of (E) peak current amplitude and (F) rate of receptor deactivation. Model deactivation kinetics τoff measured by the weighted time constant. (G, H) Progression of activation, desensitization, and deactivation of currents produced by the model and normalized by maximum amplitudes during the pulse protocol in the presence of 1 μM (G) and 10 μM (H) IVM. Insets show the magnified desensitization phases of the response. All experimental data are derived from [20]. (TIF) [file pcbi.1005643.s012.tif]

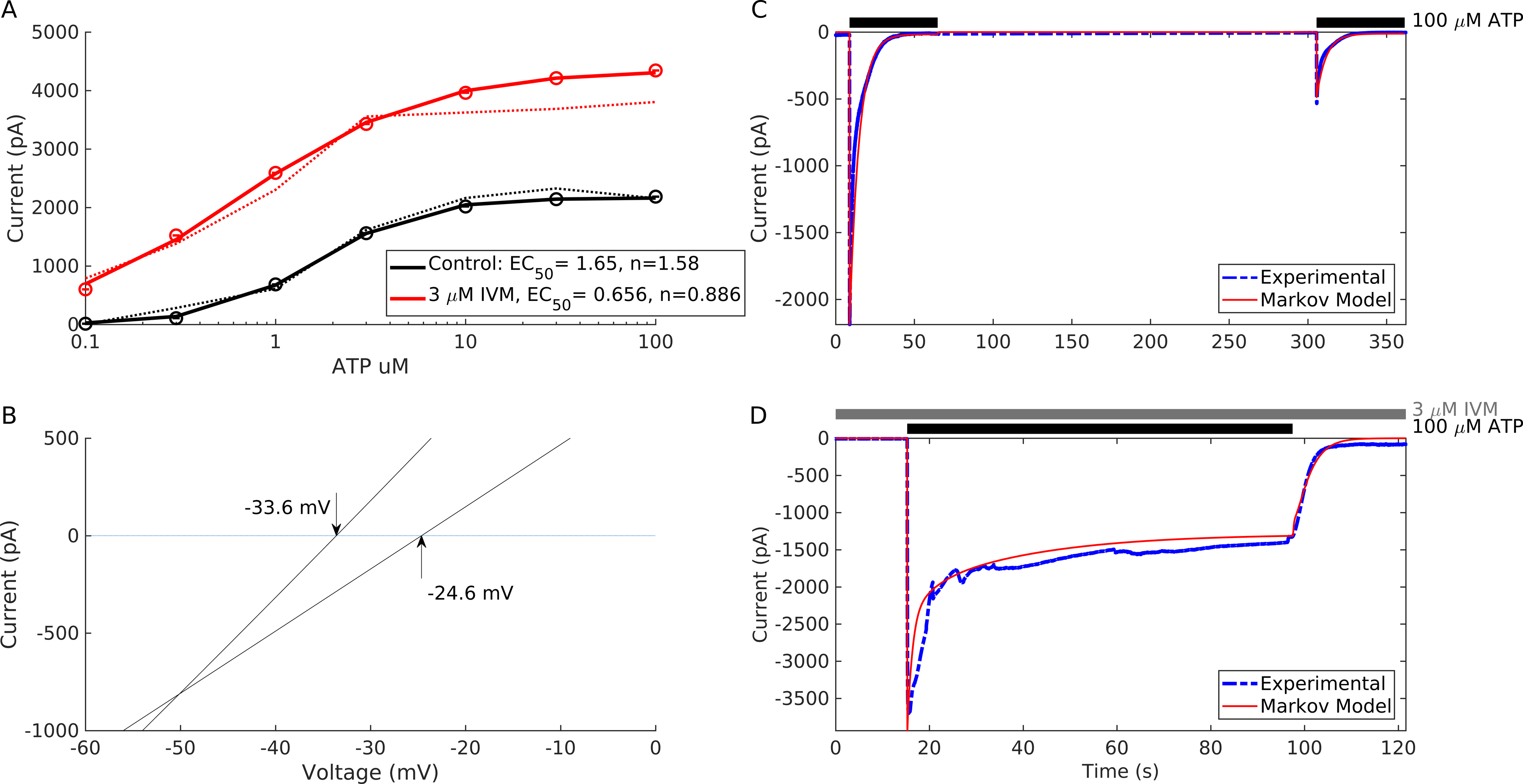

Supplement: S12 Fig — (A) ATP-dependent concentration-response curves in the absence (black) and presence (red) of 3 μM IVM applied 30 s before ATP stimulation. Calculated EC50 and Hill coefficients in the absence of IVM were 1.6549 μM and 1.5777, respectively, and in the presence of IVM were 0.65606 and 0.88552 μM, respectively. Dotted lines are experimental data. (B) The first and last I-V curves showing the decrease in their slopes and a positive shift in reversal potential upon stimulation with 100 μM ATP for 10 s in the presence of 3 μM IVM applied 20 s before ATP stimulation. The bath medium had Na+ replaced by NMDG+ modeled by setting E1 = −107.8 mV and E2 = −12.3 mV. Voltage was ramped from −80 mV to +80 mV twice per second from a holding potential of −60 mV. (C) Two prolonged applications of 100 μM ATP separated by a 4 min washout period in the absence of IVM. (D) Prolonged application of 100 μM ATP produced by the model in the presence of 3 μM IVM. All experimental data were derived from [20]. (TIF) [file pcbi.1005643.s013.tif]
